# Integration and validation of host transcript signatures, including a novel 3-transcript tuberculosis signature, to enable one-step multiclass diagnosis of childhood febrile disease

**DOI:** 10.1186/s12967-024-05241-4

**Published:** 2024-08-29

**Authors:** Samuel Channon-Wells, Dominic Habgood-Coote, Ortensia Vito, Rachel Galassini, Victoria J. Wright, Andrew J. Brent, Robert S. Heyderman, Suzanne T. Anderson, Brian Eley, Federico Martinón-Torres, Michael Levin, Myrsini Kaforou, On behalf of UK Kawasaki Disease Genetics, ILULU, GENDRES and EUCLIDS consortia, Jethro A. Herberg

**Affiliations:** 1 Section of Paediatric Infectious Disease, Department of Infectious Disease, Imperial College London, London, UK; 2 Centre for Paediatrics and Child Health, Imperial College London, London, UK; 3 Oxford University Hospitals NHS Foundation Trust, Headley Way, Headington, Oxford, UK; 4 Nuffield Department of Medicine, University of Oxford, Oxford, UK; 5 Research Department of Infection, Division of Infection and Immunity, University College London, London, UK; 6 MRC Clinical Trials Unit, University College London, London, UK; 7 Department of Paediatrics and Child Health, Faculty of Health Sciences, University of Cape Town, Cape Town, South Africa; 8 Translational Pediatrics and Infectious Diseases, Department of Pediatrics, Hospital Clínico Universitario de Santiago de Compostela, Santiago de Compostela, Galicia Spain; 9 Genetics, Vaccines, Infections and Pediatrics Research Group (GENVIP), Instituto de Investigación Santiaria de Santiago, Universidade de Santiago de Compostela, Santiago de Compostela, Spain; 10 Centro de Investigación Biomédica en Red de Enfermedades Respiratorias (CIBER-ES), Instituto de Salud Carlos III, Madrid, Spain

**Keywords:** Gene expression, Diagnostics, Kawasaki disease, Tuberculosis, Bacterial infection, Viral infection, Multiclass diagnostics

## Abstract

**Background:**

Whole blood host transcript signatures show great potential for diagnosis of infectious and inflammatory illness, with most published signatures performing binary classification tasks. Barriers to clinical implementation include validation studies, and development of strategies that enable simultaneous, multiclass diagnosis of febrile illness based on gene expression.

**Methods:**

We validated five distinct diagnostic signatures for paediatric infectious diseases in parallel using a single NanoString nCounter® experiment. We included a novel 3-transcript signature for childhood tuberculosis, and four published signatures which differentiate bacterial infection, viral infection, or Kawasaki disease from other febrile illnesses. Signature performance was assessed using receiver operating characteristic curve statistics. We also explored conceptual frameworks for multiclass diagnostic signatures, including additional transcripts found to be significantly differentially expressed in previous studies. Relaxed, regularised logistic regression models were used to derive two novel multiclass signatures: a mixed One-vs-All model (MOVA), running multiple binomial models in parallel, and a full-multiclass model. In-sample performance of these models was compared using radar-plots and confusion matrix statistics.

**Results:**

Samples from 91 children were included in the study: 23 bacterial infections (DB), 20 viral infections (DV), 14 Kawasaki disease (KD), 18 tuberculosis disease (TB), and 16 healthy controls. The five signatures tested demonstrated cross-platform performance similar to their primary discovery-validation cohorts. The signatures could differentiate: KD from other diseases with area under ROC curve (AUC) of 0.897 [95% confidence interval: 0.822–0.972]; DB from DV with AUC of 0.825 [0.691–0.959] (signature-1) and 0.867 [0.753–0.982] (signature-2); TB from other diseases with AUC of 0.882 [0.787–0.977] (novel signature); TB from healthy children with AUC of 0.910 [0.808–1.000]. Application of signatures outside of their designed context reduced performance. In-sample error rates for the multiclass models were 13.3% for the MOVA model and 0.0% for the full-multiclass model. The MOVA model misclassified DB cases most frequently (18.7%) and TB cases least (2.7%).

**Conclusions:**

Our study demonstrates the feasibility of NanoString technology for cross-platform validation of multiple transcriptomic signatures in parallel. This external cohort validated performance of all five signatures, including a novel sparse TB signature. Two exploratory multi-class models showed high potential accuracy across four distinct diagnostic groups.

**Supplementary Information:**

The online version contains supplementary material available at 10.1186/s12967-024-05241-4.

## Background

Children with fever represent one of the commonest presentations to healthcare professionals [1, 2]. However, available diagnostic tests are poor at rapid and accurate discrimination of the aetiology of fever. This limits the potential to give the right treatment to the right patient at the right time [3]. Many infectious and inflammatory disorders, including tuberculosis (TB) or Kawasaki Disease (KD), present with signs and symptoms that overlap with other conditions, and diagnosis is typically delayed until after initial management strategies for more common bacterial and viral infections fail [4, 5]. Several blood transcript-based diagnostic signatures have been published [6–8], but translation into clinically useable tests lags behind.

**Fig. 1:**
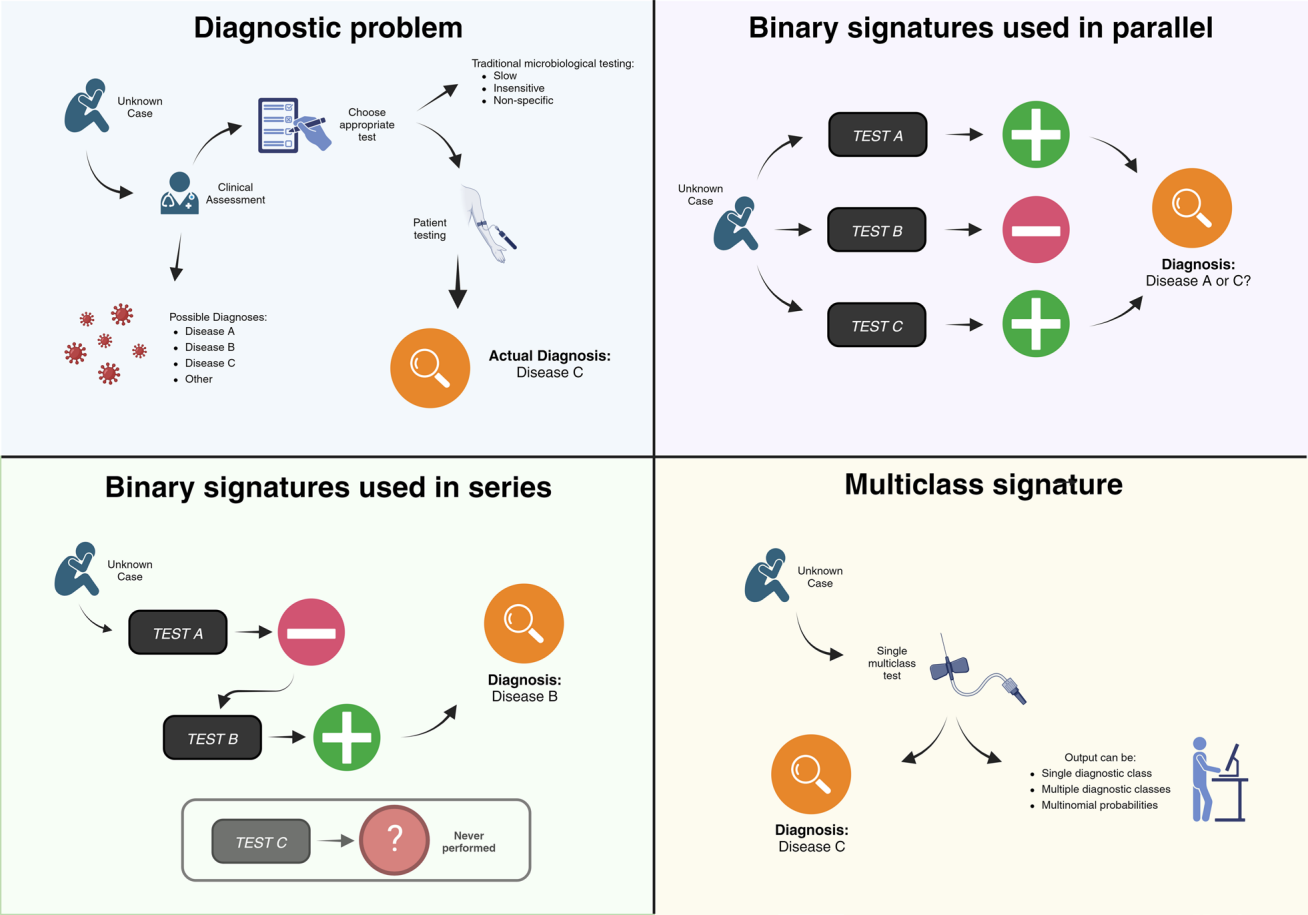
Approaches to Diagnostic testing. **A** Simple schematic approach to clinical diagnostics, with reliance on traditional microbiological testing. **B** Application of binary diagnostic signatures in parallel, demonstrating problems relating to overlapping or contradictory results. **C** Application of binary signatures in series, demonstrating carry-forward error in classification. **D** Application of multiclass signature, avoiding these limitations. Created with BioRender.com

Most existing transcriptomic diagnostic signatures published to date are binary: either one-vs-all (OVA—e.g. Kawasaki Disease versus other diseases) [9] or one-vs-one (e.g. bacterial versus viral) [10]. These show great diagnostic accuracy in many settings and are well suited to rule-in or rule-out diagnostic dilemmas. However, they are less useful for clinical presentations with diagnostic uncertainty and multiple potential differential diagnoses (Fig. 1A). Application of multiple transcriptomic signatures either in parallel or in series may simulate this process more closely, but their pairwise independence can lead to failure through multiple mechanisms (Fig. 1B, C). A single multiclass signature for the same set of diagnoses might overcome many of these limitations (Fig. 1D), accounting for the dependence of one diagnosis on the other differentials under consideration, whilst utilising the interdependence between transcripts. Indeed, Habgood-Coote et al. recently demonstrated a 161-transcript signature that can differentiate 18 acute paediatric diseases in parallel [11].

One obstacle in test development is validation of diagnostic signatures that have been discovered using methodology unsuitable for clinical translation, such as microarrays or RNA-sequencing. NanoString technology has been used for quantification of transcripts in many sample-types, enabling its use in transcriptomic signature discovery and validation [12–15], in cancer prognostics, and companion diagnostics [16–18]. We used NanoString to demonstrate the feasibility of validating multiple whole-blood binary-classification transcriptomic signatures in parallel, including a previously unpublished novel 3-transcript signature that differentiates active tuberculosis from other febrile illnesses. Efficiently parallelising validation studies in this way could substantially reduce both time and financial costs.

We also developed two multiclass diagnostic signatures, to explore proof-of-concept computational methodologies that could be applied to multiclass prediction problems in clinical diagnostics. The first model explores multiple OVA signatures in parallel, whereas the second considers all diagnostic categories simultaneously.

## Methods

### Study design and population

We performed a validation study using a subset of prospectively recruited patients from five distinct paediatric (age < 19 years) cohorts. Patients with comorbidities known to significantly affect gene expression (bone marrow transplant, immunodeficiency, or immunosuppressive treatment) were excluded. We included patients with definite bacterial (DB) or definite viral (DV) infection; Kawasaki disease (KD); or tuberculosis disease (TB). Healthy control samples were also included to improve normalisation protocols and provide context for “normal” transcript levels. All participants were independent from the derivation cohorts of the signatures we evaluated. Clinical data and samples were identified only by study number.

Disease groups were assigned using pre-agreed definitions for each primary study after review of all available clinical and laboratory data. Patients were classified as having a DB infection if a pathogenic bacterium was isolated from a normally sterile site, matching the clinical syndrome at presentation, with or without concurrent viral pathogens detected. A diagnosis of DV infection was made if a pathogenic virus was identified alongside a matching clinical syndrome, without coexisting features of bacterial infection, and with low inflammatory markers (C-Reactive Protein (CRP) < 60mg/L and absolute neutrophil count < 12,000/μL) [19]. Patients were diagnosed with complete or incomplete KD based on the 2017 American Heart Association (AHA) criteria [20]. Assignment to the tuberculosis disease group required a clinical history suggestive of TB and corroborative laboratory testing (culture for *M. tuberculosis*, Interferon-Gamma Release Assays, or positive tuberculin skin test). TB patients with coincident HIV were excluded. In all groups, samples were collected as soon as possible after presentation, and wherever possible before initiation of relevant treatment. Additional details, including full inclusion and exclusion criteria, are described in the Additional file 1 and original papers [10, 21–23].

### Ethical approvals

Written informed consent was obtained from parents or guardians using locally approved research ethics committee permissions (Ethical Committee of Clinical Investigation of Galicia (GENDRES CEIC ref 2010/015); UK National Research Ethics Service (UK Kawasaki Genetics 13/LO/0026; EUCLIDS 11/LO/1982; NIKS 11/11/11)). Patients in Cape-Town (ILULU) were recruited under ethics approvals from the local recruiting centre: HREC REF 130/2007 [22].

### Selected signatures

Five whole-blood-based RNA signatures that differentiate febrile diseases were selected for validation using NanoString: These signatures are:

- A 13-transcript signature to distinguish KD from other febrile illnesses [9], previously validated via RT-PCR [24]. (Wright13)
- A 2-transcript signature to distinguish Bacterial from Viral infections in children [10], previously validated via RT-PCR [25, 26]. (Herberg2)
- A 2-transcript signature adapted from Herberg2 signature, with *FAM89A* substituted for the highly correlated but more abundantly expressed transcript *EMR1-ADGRE1* [27]. (Pennisi2)
- A single transcript signature, *BATF2*, to distinguish TB disease from healthy adults [28], externally validated in RNAseq and microarray datasets [29–31]. (BATF2)
- An unpublished 3-transcript signature to distinguish TB disease from other diseases (TB3)

The target diseases for the signatures were selected to represent a diverse range of important causes of fever in children. The two bacterial-viral signatures (Herberg2 and Pennisi2) were selected for further cross-platform validation, and to assess the effect on performance of the substitution of *FAM89A* for *EMR1-ADGRE1*. We selected the 13-transcript signature for KD as this is an important cause of fever in children, that is often misdiagnosed as an infectious disease, and allows us to demonstrate our approach on an important inflammatory disorder. The TB3 signature was included to provide the first cross-platform validation of this novel signature. We compared TB3 to a primarily adult signature (*BATF2*) for TB disease to investigate the performance of this adult-derived signature in children, and to characterize its performance when applied to a new task of differentiating TB disease from other causes of fever.

Derivation of the TB3 signature is described in more detail in Additional file 1 and results. Briefly, the signature was generated by randomly splitting the discovery cohort described in the Anderson et al. study [22] into training (80%) and test sets (20%), and running Forward Selection-Partial Least Squares on the training set [10, 32].

### Transcript selection

A total of 69 transcripts were selected to be run in the Nanostring panel (Table S1). All 20 transcripts from the five validation signatures were included. We selected an additional 40 transcripts that have previously been found to accurately discriminate between one or more of the above comparator conditions in RNA-sequencing or microarray data (Table S1). Selected transcripts include predominantly those associated with protein coding genes, such as the Type 1 interferon stimulated gene *IFI44L* (Interferon-Induced Protein 44-Like), which has previously been implicated in response to viral infections [10]. Smaller numbers of transcripts are associated with long non-coding RNAs (lncRNAs) or microRNAs. Examples include the lncRNA *KLF7-IT1* (Kruppel-Like Factor 7 Intronic Transcript 1) and *MIR3128* (microRNA 3128), which have no previous recorded disease associations [33], but were both differentially expressed between bacterial and viral infections in the work of Habgood-Coote et al [11].

We also included three housekeeping transcripts recommended by NanoString, and six more, identified from our microarray and RNA-sequencing data, that had the smallest standard-deviation/mean ratio (coefficient of variation) across multiple separate cohorts for different expression abundance ranges.

### Sample and data processing

Total RNA was extracted from whole blood from PAXgene tubes using PAXgene Blood miRNA kits (PreAnalytiX), and transcript expression quantification was undertaken with 100 ng of RNA using the NanoString nCounter® MAX system, and a custom designed codeset of the selected transcripts. Raw counts were normalised and log-transformed (Additional file 1).

### Statistical analyses

All statistical analyses were undertaken in R, version 4.1.1 [34].

#### Descriptive statistics and signature evaluation

The diagnostic accuracy of each signature was calculated as an area under receiver operator characteristic curve (AUC) with 95% confidence intervals (CI), using the DeLong method in the R-package pROC [35, 36]. pROC was used for plotting receiver operator characteristic (ROC) curves with 95% confidence intervals of the sensitivities at fixed specificities. The optimal threshold was chosen to maximise the Youden’s J statistic [37], and then used to calculate additional test statistics with 95% CI, including sensitivity and specificity, using the R-package epiR [38]. A disease risk score (DRS) was calculated for each signature by summation of up-regulated transcripts and subtraction of down-regulated transcripts on a logarithmic scale, as previously described by Kaforou et al. [39]. A logistic regression was refitted on log-scale normalised counts for each signature to retrain coefficients and derive prediction probabilities.

#### Multiclass prediction models

We chose two distinct models to predict from one of four diseases (DB, DV, KD & TB). The Mixed One-vs-All (MOVA) model optimises four binary OVA models in parallel, one for each disease. The Multiclass model performs multivariate logistic regression. Full descriptions are available in Additional file 1. Both methods use relaxed, regularised binomial/multinomial logistic regression models (elastic net) [40], implemented using the R-package glmnet [41], to account for the large number of predictors relative to samples and inherent multicollinearity in our data. Healthy controls were removed, and samples were weighted by group size to account for class imbalance. The original 60 transcripts were restricted to a subset of 36 that were significantly different for one or more one-vs-all comparisons, using a Mann–Whitney U test, with correction for multiple hypothesis testing. This criterion was applied to remove transcripts performing poorly when moving cross-platform—typically lowly expressed transcripts—enabling a refining of the feature space to more relevant transcripts. In-sample error rates and confusion matrices are reported for both models.

## Results

### Participants

**Fig. 2:**
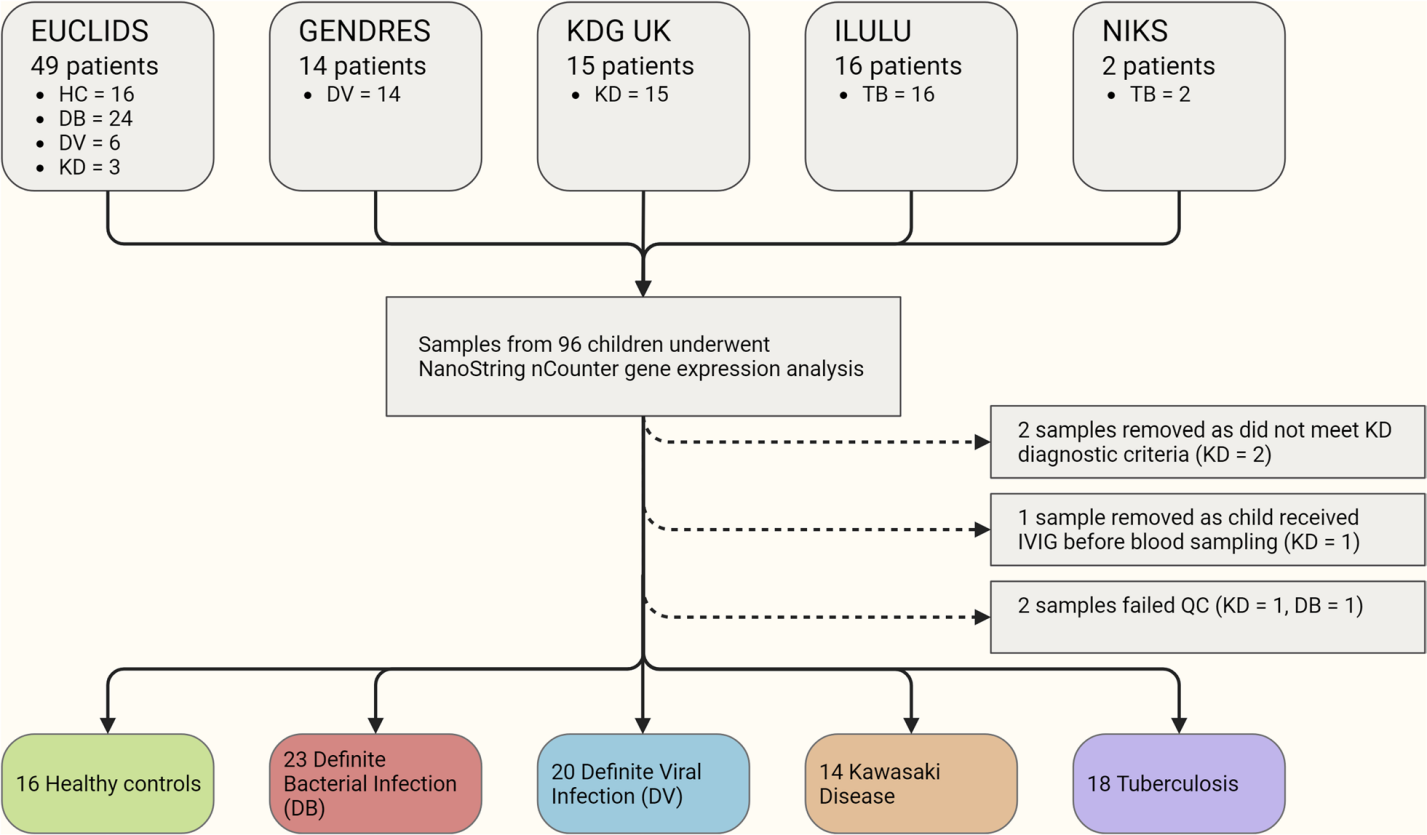
Study overview. Schematic overview of study recruitment and analysis. Created with BioRender.com

Samples from 91 children were included in this study: 16 healthy controls, 23 with DB, 20 DV, 14 KD and 18 TB (Fig. 2). One KD patient was removed who received IVIG before blood sampling, and two were removed after transcript expression quantification following blinded review of clinical data, as they did not meet AHA criteria for complete or incomplete KD. We excluded 2 samples due to low expression levels after quality control (Additional file 1). Baseline demographic and clinical data are shown in Table 1. DB and DV patients had similar demographics, whereas healthy controls were older than other patients. KD and TB patients were generally older and were less likely to be of European ethnicity. Ethnicity data for TB patients recruited in Cape Town were not collected in the index study.

**Table 1:** Patient characteristics

	Controls	Definite bacterial	Definite viral	Kawasaki disease	Tuberculosis
Number of Patients	16	23	20	14	18
Female, %	50	52.2	50	28.6	38.9
Median age, years [IQR] (missing, n)	6.1 [3.0–11.0] (0)	1.2 [0.5–3.5] (0)	1.0 [0.2–2.3] (0)	2.4 [0.8–3.8] (0)	2.6 [1.4–4.8] (0)
ETHNICITY					
African/North African	3	3	1	1	0
Asian	3	1	0	4	0
Black African	0	0	0	4	2
European	7	15	15	5	0
South American	0	2	0	0	0
Other/Mixed	3	2	4	0	0
Unknown	0	0	0	0	16
Median CRP, mg/L [IQR] (missing, n)	0.0 [0.0–0.0] (15)	99.0 [34.8–189.2] (3)	23.8 [20.0–32.0] (13)	109.3 [90.0–163.0] (1)	13.5 [11.8–15.2] (16)
Median WBC, × 10^9^/L [IQR] (missing, n)	10.6 [7.8–11.7] (10)	13.2 [6.1–25.0] (0)	10.7 [7.8–16.3] (7)	17.0 [13.1–19.2] (0)	10.1 [7.2–20.9] (3)
Median Neutrophils, × 10^9^/L [IQR] (missing, n)	3.4 [2.9–4.8] (10)	10.4 [4.2–20.4] (2)	4.5 [3.6–7.6] (9)	12.9 [9.1–15.9] (0)	6.5 [3.1–11.3] (5)
Median Lymphocytes, × 10^9^/L [IQR] (missing, n)	4.8 [3.1–6.4] (10)	2.1 [1.0–4.1] (2)	3.7 [2.8–5.4] (9)	2.2 [1.2–3.0] (0)	3.6 [1.9–5.0] (5)
PICU admissions % (missing, n)	NA	56.5 (0)	20.0 (0)	7.1 (0)	5.6 (0)
Days from symptom onset* to sample collection [IQR] (missing, n)	NA	4.5 [3.0–7.8] (1)	5.0 [5.0–6.0] (11)	6.0 [5.0–8.5] (0)	NA (18)
Days from hospital admission to sample collection [IQR] (missing, n)	NA	1.0 [0.0–1.0] (0)	0.0 [0.0–1.0] (1)	1.0 [0.2–1.8] (0)	NA (18)

Demographic and clinical characteristics of patients included

*CRP* C-reactive protein, *IQR* Interquartile range, *PICU* paediatric intensive care unit, *WBC* White blood cells

*Symptom onset for Kawasaki disease patients defined as onset of fever

Pathogens identified in infected patients are presented in Table S2. Admission-to-sample collection times were short, with median of < 2-days for all groups where data were available. Median days from fever-onset to sampling in KD patients was 6 (IQR 5–9) and was similar to the symptom onset to sample collection time in DB and DV patients. Diagnostic performance of routinely-measured CRP and White Blood Cell, Neutrophil, and Lymphocyte counts for binary and one-vs-all comparisons are shown in Table S3.

### Signature validation

#### Kawasaki 13-transcript signature

**Fig. 3:**
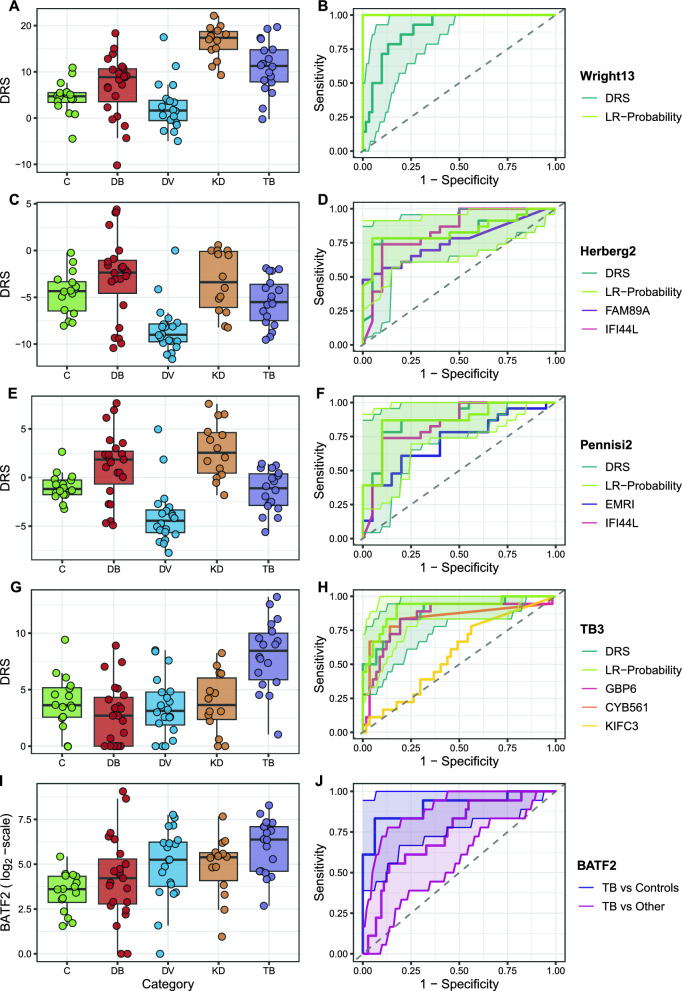
Performance of existing signatures. Plots of Disease Risk Scores by category (left) and ROC-curves (right) for five signatures. **A** and **B** Wright13 signature, with boxplots of the DRS by category **A** and ROC curves of the DRS and LR-probability (**B**). **C** and **D** Herberg2 signature, with boxplots of the DRS by category **C** and ROC curves of the DRS, LR-probability, and individual transcripts (**D**). **E** and **F** Pennisi2 signature, with boxplots of the DRS by category (**E**) and ROC curves of the DRS, LR-probability, and individual transcripts (**F**). **G** and **H** TB3 signature, with boxplots of the DRS by category (**G**) and ROC curves of the DRS, LR-probability, and individual transcripts (**H**). **I** and **J** BATF2, with boxplots of expression by category (**I**) and ROC-curves of BATF2 expression tasked with differentiating active TB from either controls or other disease groups **J**. 95% confidence intervals for ROC-curves are included for the DRS and LR-probability only in panels **B**, **D**, **F** and **H**

The Wright13 DRS was able to diagnose KD from other diseases with high accuracy (Fig. 3A). ROC curve analysis demonstrated an AUC of 0.897 (95% Confidence interval 0.822–0.972, Table 2), with optimal sensitivity of 0.929 (0.661–0.998) and specificity of 0.738 (0.609–0.842) (Fig. 3B). As observed previously, the DRS was more discriminatory earlier in the disease course of KD patients (Figure S1). Refitting a logistic regression model using all 13 transcripts had 100% accuracy to diagnose KD (Table 2, Fig. 3B).

**Table 2:** Diagnostic accuracy of each signature

	AUC	Sensitivity	Specificity	Positive likelihood ratio	Negative likelihood ratio
Wright13 DRS—KD vs other	0.897 [0.822–0.972]	0.929 [0.661–0.998]	0.738 [0.609–0.842]	3.540 [2.268–5.526]	0.097 [0.015–0.644]
Wright13 LR score—KD vs other	1.000 [NA]*	1.000 [0.768–1.000]	1.000 [0.941–1.000]	119.867 [7.570–1898.060]	0.034 [0.002–0.513]
Herberg2 DRS—DB vs DV	0.825 [0.691–0.959]	0.739 [0.516–0.898]	0.950 [0.751–0.999]	14.783 [2.155–101.408]	0.275 [0.137–0.550]
Herberg2 LR score—DB vs DV	0.838 [0.711–0.965]	0.783 [0.563–0.925]	0.950 [0.751–0.999]	15.652 [2.289–107.024]	0.229 [0.105–0.500]
Herberg2 DRS—DB vs other	0.699 [0.556–0.842]	0.739 [0.516–0.898]	0.769 [0.632–0.875]	3.203 [1.843–5.565]	0.339 [0.168–0.686]
Herberg2 LR score—DB vs other	0.723 [0.581–0.866]	0.783 [0.563–0.925]	0.692 [0.549–0.813]	2.543 [1.604–4.033]	0.314 [0.142–0.696]
Herberg2 DRS—DV vs other	0.844 [0.739—0.948]	0.900 [0.683–0.988]	0.764 [0.630–0.868]	3.808 [2.316–6.259]	0.131 [0.035–0.492]
Herberg2 LR score—DV vs other	0.849 [0.747–0.951]	0.900 [0.683–0.988]	0.764 [0.630–0.868]	3.808 [2.316–6.259]	0.131 [0.035–0.492]
Pennisi2 DRS—DB vs DV	0.867 [0.753–0.982]	0.783 [0.563–0.925]	0.900 [0.683–0.988]	7.826 [2.065–29.659]	0.242 [0.110–0.532]
Pennisi2 LR score—DB vs DV	0.872 [0.760–0.984]	0.870 [0.664–0.972]	0.900 [0.683–0.988]	8.696 [2.313–32.691]	0.145 [0.050–0.421]
Pennisi2 DRS—DB vs other	0.696 [0.568–0.825]	0.696 [0.471–0.868]	0.692 [0.549–0.813]	2.261 [1.386–3.687]	0.440 [0.231–0.837]
Pennisi2 LR score—DB vs other	0.696 [0.567–0.826]	0.739 [0.516–0.898]	0.635 [0.490–0.764]	2.023 [1.312–3.118]	0.411 [0.200–0.843]
Pennisi2 DRS—DV vs other	0.865 [0.755–0.974]	0.900 [0.683–0.988]	0.782 [0.650–0.882]	4.125 [2.450–6.946]	0.128 [0.034–0.480]
Pennisi2 LR score—DV vs other	0.872 [0.773–0.970]	0.900 [0.683–0.988]	0.873 [0.755–0.947]	7.071 [3.486–14.344]	0.115 [0.031–0.428]
TB3 DRS—TB vs other	0.882 [0.787–0.977]	0.833 [0.586–0.964]	0.807 [0.681–0.900]	4.318 [2.443–7.633]	0.207 [0.073–0.585]
TB3 LR score—TB vs other	0.914 [0.830–0.999]	0.944 [0.727–0.999]	0.825 [0.701–0.913]	5.383 [3.033–9.556]	0.067 [0.010–0.454]
BATF2—TB vs healthy controls	0.910 [0.808–1.000]	0.833 [0.586–0.964]	0.938 [0.698–0.998]	13.333 [1.976–89.946]	0.178 [0.063–0.503]
BATF2—TB vs other	0.743 [0.620–0.866]	0.556 [0.308–0.785]	0.863 [0.762–0.932]	4.056 [1.996–8.238]	0.515 [0.305–0.870]

Diagnostic accuracy statistics for primary comparisons of each signature

*AUC* Area under receiver operator characteristic curve

*Unable to calculate confidence interval for AUC using DeLong method when AUC exactly equal to one

#### Bacterial vs Viral 2-transcript signatures

For differentiating DB from DV cases the Herberg2 DRS had an AUC of 0.825 (0.691–0.959, Table 2), with sensitivity of 0.739 (0.516–0.898) and specificity of 0.950 (0.751–0.999) (Fig. 3C). There was marginal improvement after retraining coefficients using logistic regression, which was not statistically significant (p = 0.392, Fig. 3D). Both models performed significantly worse when tasked with differentiating DB from all other disease groups, with AUCs of 0.699 and 0.723 respectively, but were excellent at differentiating DV from all other disease groups, with AUCs of 0.844 and 0.849. This discrepancy may be explained by the high AUC of *IFI44L* in differentiating DV from other diseases (0.834), compared with the low AUCs (all < 0.7) of *IFI44L* for DB-vs-other diseases and *FAM89A* for both comparisons (Table S4).

As previously seen, *FAM89A* had low expression in most samples, whereas *EMR1-ADGRE1* showed more robust expression levels (Table S4). The Pennisi2 DRS replaces *FAM89A* in Herberg2 with *EMR1-ADGRE*, which improved the overall signature AUC to 0.867 (0.753–0.982) (Fig. 3E, F), although this was non-significant (p = 0.417). However, *EMR1-ADGRE1* had a lower AUC than *FAM89A* for differentiating DB and DV cases (0.717 vs 0.761, p = 0.636), suggesting the improved performance of the Pennisi2 signature is due to improved transcript interactions, rather than better performance of individual transcripts. Similar to Herberg2, the Pennisi2 signature was more accurate at differentiating viral infections from other diseases than bacterial infections vs other diseases (Table 2).

#### Tuberculosis signatures

##### Performance in microarray dataset

**Fig. 4:**
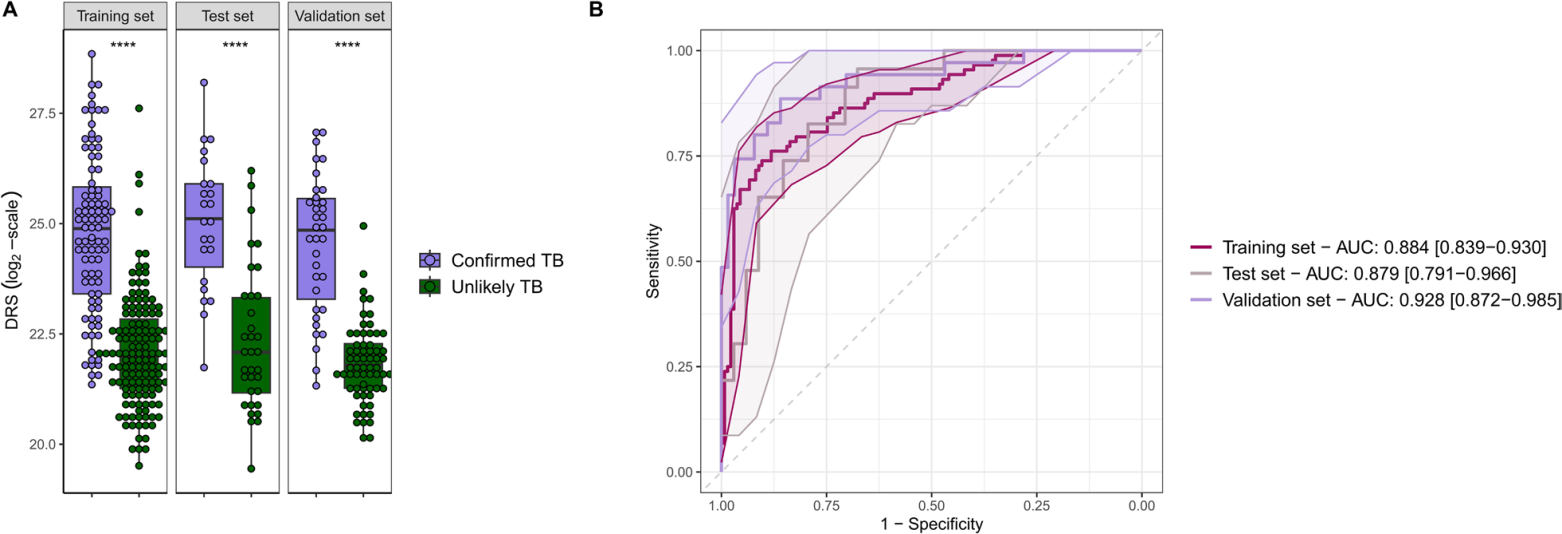
TB3 signature performance in microarray training and validation cohorts. Boxplots of the DRS **A** of the TB3 signature and the correspondent ROC curves **B** in the training, test, and validation sets

The novel TB3 signature includes the transcripts *CYB561, GBP6* and *KIFC3*. It achieved an AUC of 0.928 (0.872–0.985) in the validation cohort, with optimal sensitivity and specificity of 0.886 (0.771–0.971) and 0.859 (0.766–0.938) respectively (Additional file 1: Table S5, Fig. 4).

##### Performance in NanoString dataset

The 3-transcript TB signature was able to differentiate TB from other diseases with an AUC of 0.882 (0.787–0.977, Table 2), with sensitivity of 0.833 (0.586–0.964) and specificity of 0.807 (0.681–0.900). Again, retraining using logistic regression demonstrated only marginal improvements (Table 2, Fig. 3G, H), without statistical significance (p = 0.114).

*BATF2* alone could accurately differentiate active TB from healthy controls (AUC of 0.910 (0.808–1.000), Table 2), with high specificity, 0.938 (0.698–0.998), and sensitivity of 0.833 (0.586–0.964). However, *BATF2* was also overexpressed in patients with other diseases (Fig. 3I, J) and had significantly reduced diagnostic accuracy comparing TB with other disease groups (AUC 0.743 (0.620–0.866), p = 0.043).

### Expression patterns of individual transcripts

AUCs and summary statistics for transcript-specific one-vs-all disease comparisons are shown in Additional file 1: Table S3. The highest AUCs were found for transcripts distinguishing DV or TB from other disease groups (Additional file 1: Figure S2).

When ranked by p-value (corrected for multiple testing), 50 transcript-specific one-vs-all comparisons were significant at the alpha = 0.1 level, including 36 unique transcripts shared approximately evenly across comparisons (Additional file 1: Table S4). Two transcripts, *KLHL2* & *IFI27*, contributed to three separate significant comparisons.

### MOVA and multiclass model prediction results

**Fig. 5:**
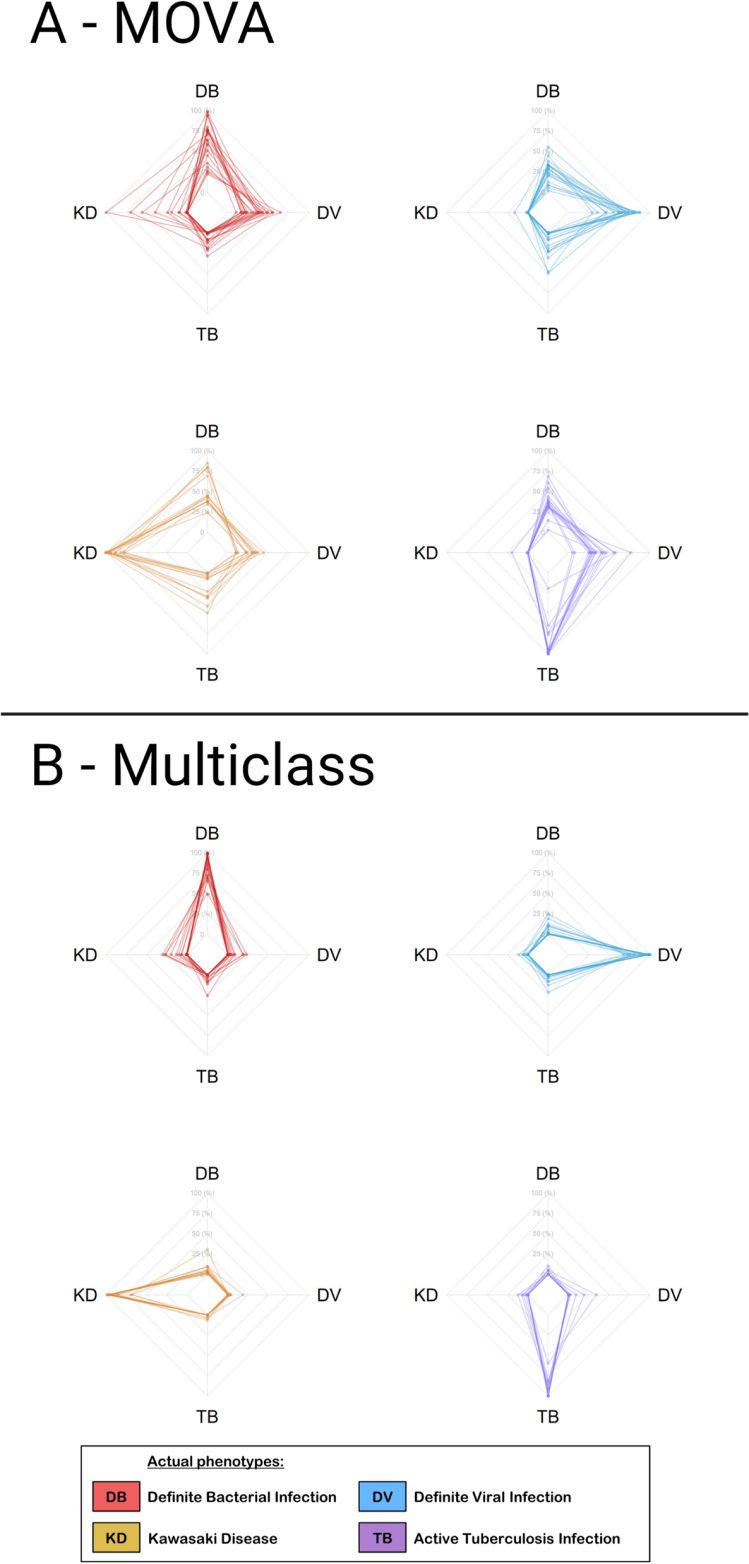
In-sample radar plots for multiclass signatures. Radar plots showing the probabilities of each class predicted by **A** the MOVA-model, **B** the Multiclass model. Probabilities separated and coloured by actual disease: Red = DB; Blue = DV; Purple = TB; Yellow = KD

When the MOVA-model was used for prediction, it selected 25 unique transcripts across four separate binomial models (Additional file 1: Figure S3), with a maximum of 9-transcripts in any single model. The MOVA-model had an in-sample error rate across all diseases of 13.3%, with worst performance predicting DB. The in-sample error rates of the separate models varied from 2.7% for TB vs other diseases to 18.7% for DB vs other diseases (Additional file 1: Table S6). Prediction probabilities for DB and KD patients were similar to each other, whereas TB patients often had high prediction probabilities for DV (Fig. 5A).

The Multiclass model selected 20 unique transcripts and had 100% prediction accuracy (Additional file 1: Table S6). 17 transcripts overlapped with the MOVA-model. In contrast to the MOVA-model, radar plots show the prediction probabilities are near one for the correct disease for all samples (Fig. 5B). When assessed on healthy controls both models predicted nearly half of patients to have TB, with the Multiclass model classifying more of the remaining cases as DB, whilst the MOVA-model classified most of the remaining controls as DV (Additional file 1: Table S6).

## Discussion and conclusion

We have shown that validation of multiple transcriptomic signatures can be efficiently performed through a single NanoString assay. The performance of all tested signatures was similar to that of their primary studies, suggesting that both the signatures and technology are robust to alterations in study design and methodology. This also implies that overfitting to discovery cohorts was not an issue for any of the tested signatures. Further evidence for this was seen by the minimal gains in performance when retraining models using logistic regression, with the exception of the larger KD signature, although this improvement may represent overfitting. Transcriptomic signatures are rapidly expanding, both in scope and number. To understand how best to implement the increasingly complex list of published transcriptomic signatures in clinical practice, new methods are needed that enable efficient evaluation of multiple signatures simultaneously. We have used NanoString to measure transcript abundances covering multiple binary signatures, to facilitate the side-by-side comparison of signature performance, and to consider alternative methodologies for assigning disease class.

A limitation of transcriptome-derived diagnostic signatures is the study-specific bias of transcripts selected by a single methodology and patient cohort [42]. This can lead to reduced performance when signatures are applied to external datasets, or different clinical settings where the case-mix is dissimilar to the discovery cohort [42, 43]. The second phenomenon was evident in our data, for example, the *BATF2* transcript performed well in its designed classification task (distinguishing TB from healthy controls) but performed poorly when differentiating TB from other diseases.

The major mechanisms behind such reduced performance in new cohorts are (1) overfitting of the original classification model, (2) under-representative discovery cohorts which do not reflect the clinical variability in the target population, or (3) failure in translation between technologies. The first mechanism can be addressed through thoughtful machine-learning pipelines in signature development, using appropriate test, training, and validation sets. However, mitigation of the second mechanism requires clinical recruitment that is representative of the full range of patient pathologies, rather than a restricted set of target conditions. Deriving binary signatures from these more varied cohorts may be clinically valid in certain contexts, e.g., for patients with specific diseases where confirmatory early diagnosis is needed. Kawasaki disease, for example, commonly presents with a cluster of characteristic clinical features, but diagnosis is frequently delayed due to clinical overlap with other conditions. A signature to diagnose or exclude KD in patients presenting with a KD-like features could improve time-to-diagnosis and outcomes [44].

However, this binary approach is inappropriate for undifferentiated febrile illness, where a wider range of pathologies are present. Multiclass models to classify patients into one or more of many possible outcomes may be the most parsimonious solution. They could enable one-step diagnosis of a range of conditions including those not considered by the physician, improving diagnostic accuracy, and reducing time-to-diagnosis.

Our exploratory analysis of multiclass diagnostic methods demonstrates the potential of this approach using a small dataset generated with techniques that are closer to patient translation than the transcriptomic approaches used to generate data for signature discovery. Our findings are supported by large-scale in silico studies based on transcriptomic data [11]. We demonstrate that two contrasting approaches—MOVA and multiclass—can both yield high in-sample classification accuracy. The MOVA model had slightly worse performance compared to the multiclass model, which had perfect in-sample accuracy. This may be explained by the restriction imposed on the MOVA model to use only certain transcripts for each comparison. However, this also exposes the multiclass model to greater risk of overfitting. Larger studies are needed to test a broader range of conceptual frameworks and methodologies, and to assess the robustness of these two exploratory models.

One unique aspect of this work is the exploration of these two models, which use different approaches to handle the potential for gene-expression patterns to overlap between multiple disorders. The MOVA-model combines four binary models, each of which is optimised for a single one-vs-all comparison. Each binary model was derived using the same initial list of 36 transcripts, so this approach has the potential to introduce redundancy into the combined final model. However, in the final penalised regression models only two transcripts out of 25 were selected for more than one binary model. This may potentially be explained by the model setup. Each binary model was trained to differentiate a single target condition from all other categories, so transcripts sharing expression patterns between two categories were unlikely to be selected by any single binary model.

In contrast, the Multiclass model directly addresses overlapping expression patterns during model training, by providing each transcript its own coefficient for each condition from the four target diagnoses. These differences in approach may provide further explanation for the performance difference between the models.

In our dataset, transcripts that have been shown to differentiate DB from DV infections were much better at differentiating viral infections from other diseases than bacterial infections from other diseases. Most transcripts performed poorly when comparing DB with other diseases: of the top 20 AUCs only one distinguishes DB from other diseases (*HP*, AUC 0.784). This is consistent with previous transcriptomic studies, which have shown viral infections are easier to distinguish from other causes of febrile illness than bacterial infections are [45, 46]. A plausible explanation for this phenomenon is the existence of highly conserved host-responses to viral infections, such as the Interferon Stimulated Genes [47], whereas host-responses to bacterial infection may be more varied, in part due to their larger and more varied genomes.

The unpublished novel 3-transcript TB signature demonstrated high sensitivity and specificity in this external cohort, with performance similar to, or exceeding that of previously published signatures [48]. The included transcripts are a subgroup of the original 51-transcript signature of Anderson et al. [22], showing in principle that reduction in transcript numbers can be achieved whilst maintaining high performance. Previous signatures developed to distinguish TB from other diseases often fail to differentiate viral infections from TB, potentially due to reliance on interferon stimulated genes [48]. The 3-transcript signature does not include interferon stimulated genes or transcripts from related pathways, which may explain its high performance despite including viral infections in the comparator group. Although non-significant, retraining the signature to the new platform using logistic regression did demonstrate improved accuracy, exceeding the WHO-defined target-product profile for triage assessment [49]. Furthermore, the sparseness of the signature may aid in translation to a clinically useable assay, whilst simultaneously minimising costs.

Clear limitations of our study include the small sample size, and use of samples from heterogeneous studies. We have attempted to address these through appropriate normalisation processes where possible. Due to its size, the study was limited to only a few diseases of interest, but we consider that our conclusions are valid for the methodological suitability of the NanoString platform for this parallel validation task. Further large-scale studies are needed to explore different conceptual frameworks for the clinical implementation of omic-based signatures, and to determine how to best integrate these novel technologies within existing clinical frameworks. Such studies should also assess the importance of multiclass model setup, for example, altering the regularisation strength for regression models, and comparing regression classifiers with other non-linear methods, such as random forest models.

Although most transcripts demonstrated measurable expression levels and good classification performance when converting from RNAseq to NanoString, some had poor detection. Loss of detection is previously described in cross-platform gene expression studies [24]. Lack of resolution for detection of low abundance transcripts using NanoString nCounter®, relative to the discovery platforms, may have reduced the utility of certain transcripts, and in future studies various input RNA quantities should be trialled to maximise transcript detection. Our findings highlight the need for cross-platform assessment of candidate diagnostic signatures, and consideration of the limitations of each methodology at the earliest stage of signature derivation.

Since CRP and neutrophil cutoffs were used for phenotyping viral patients, and CRP measurements were only available for 2 patients in the TB category, it was not possible to compare the diagnostic performance of the five validated signatures to these commonly used clinical biomarkers without confounding. However, it is known that performance of both CRP and blood cell measurements have limited combined sensitivity and specificity for causes of fever in children, and no well-defined cutoffs exist [6, 50]. The high-performance of the five validated models provides further demonstration of the potential for host-response diagnostic signatures to greatly influence clinical care in paediatrics, through improving diagnostic accuracy and reducing diagnostic delays.

Despite this, it remains the case that most existing signatures have yet to make the leap from bench to bedside. Such translation is particularly challenging for diagnostics in those presenting acutely with fever, where accurate diagnostics within a few hours is most needed [2]. In this setting, we have shown that NanoString may aid in bridging the gap between expensive untargeted gene expression quantification methods (e.g., RNAseq, microarrays) and cheaper, rapid technologies (e.g., qRT-PCR), enabling quick, cost-effective parallel evaluation and refinement of a variety of diagnostic signatures.

## Conclusions

Our cross-platform study demonstrates in principle the utility of NanoString technology for efficient parallel validation of transcriptomic signatures. Our out-of-sample findings validated five distinct signatures, including a novel sparse TB signature, but with a reduction in discriminatory power in patients drawn from outside their remit. Two exploratory multi-class models showed high accuracy across multiple disparate diagnostic groups, highlighting the potential of this approach.

### Supplementary Information

**Additional file 1: **Supplementary methods and results.

**Additional file 2: **Annex 1. Consortia members.

## Data Availability

Raw NanoString data with phenotype labels will be made available to bona fide researchers upon reasonable request to the lead contact.

## Abbreviations

AHA: American Heart Association
AUC: Area under receiver operator characteristic curve
CRP: C-reactive protein
DB: Definite bacterial
DRS: Disease risk score
DV: Definite viral
HIV: Human immunodeficiency virus
IQR: Interquartile range
IVIG: Intravenous immunoglobulin
KD: Kawasaki disease
LR: Logistic regression
MOVA: Mixed one-versus-all model
OVA: One-versus-all
PICU: Paediatric intensive care unit
ROC: Receiver operator characteristic
RT-PCR: Reverse transcription polymerase chain reaction
qRT-PCR: Quantitative real-time reverse-transcription polymerase chain reaction
TB: Tuberculosis

## References

[1] Liu L, Oza S, Hogan D, et al. Global, regional, and national causes of under-5 mortality in 2000–15: an updated systematic analysis with implications for the sustainable development goals. Lancet. 2016;388(10063):3027–35. 27839855 10.1016/S0140-6736(16)31593-8 PMC5161777

[2] Nijman RG, Jorgensen R, Levin M, Herberg J, Maconochie IK. Management of children with fever at risk for pediatric sepsis: a prospective study in pediatric emergency care. Front Pediatr. 2020;8:548154. 33042929 10.3389/fped.2020.548154 PMC7527403

[3] Martinon-Torres F, Salas A, Rivero-Calle I, et al. Life-threatening infections in children in Europe (the EUCLIDS Project): a prospective cohort study. Lancet Child Adolesc Health. 2018;2(6):404–14. 30169282 10.1016/S2352-4642(18)30113-5

[4] Moore A, Harnden A, Mayon-White R. Recognising Kawasaki disease in UK primary care: a descriptive study using the clinical practice research datalink. Br J Gen Pract. 2014;64(625):e477–83. 25071060 10.3399/bjgp14X680953 PMC4111340

[5] Lee JH, Garg T, Lee J, et al. Impact of molecular diagnostic tests on diagnostic and treatment delays in tuberculosis: a systematic review and meta-analysis. BMC Infect Dis. 2022;22(1):940. 36517736 10.1186/s12879-022-07855-9 PMC9748908

[6] Zandstra J, Jongerius I, Kuijpers TW. Future biomarkers for infection and inflammation in febrile children. Front Immunol. 2021;12:631308. 34079538 10.3389/fimmu.2021.631308 PMC8165271

[7] Leticia Fernandez-Carballo B, Escadafal C, MacLean E, Kapasi AJ, Dittrich S. Distinguishing bacterial versus non-bacterial causes of febrile illness—a systematic review of host biomarkers. J Infect. 2021;82(4):1–10. 33610683 10.1016/j.jinf.2021.01.028

[8] Ross MH, Zick BL, Tsalik EL. Host-based diagnostics for acute respiratory infections. Clin Ther. 2019;41(10):1923–38. 31353133 10.1016/j.clinthera.2019.06.007

[9] Wright VJ, Herberg JA, Kaforou M, et al. Diagnosis of Kawasaki disease using a minimal whole-blood gene expression signature. JAMA Pediatr. 2018;172(10):e182293. 30083721 10.1001/jamapediatrics.2018.2293 PMC6233768

[10] Herberg JA, Kaforou M, Wright VJ, et al. Diagnostic test accuracy of a 2-transcript host RNA signature for discriminating bacterial vs viral infection in febrile children. JAMA. 2016;316(8):835–45. 27552617 10.1001/jama.2016.11236 PMC5997174

[11] Habgood-Coote D, Wilson C, Shimizu C, et al. Diagnosis of childhood febrile illness using a multi-class blood RNA molecular signature. Med. 2023;4(9):635-54 e5. 37597512 10.1016/j.medj.2023.06.007

[12] Trouillet-Assant S, Viel S, Ouziel A, et al. Type I interferon in children with viral or bacterial infections. Clin Chem. 2020;66(6):802–8. 32359149 10.1093/clinchem/hvaa089

[13] Petrilli JD, Araujo LE, da Silva LS, et al. Whole blood mRNA expression-based targets to discriminate active tuberculosis from latent infection and other pulmonary diseases. Sci Rep. 2020;10(1):22072. 33328540 10.1038/s41598-020-78793-2 PMC7745039

[14] Bauer W, Kappert K, Galtung N, et al. A Novel 29-messenger RNA host-response assay from whole blood accurately identifies bacterial and viral infections in patients presenting to the emergency department with suspected infections: a prospective observational study. Crit Care Med. 2021;49(10):1664–73. 34166284 10.1097/CCM.0000000000005119 PMC8439671

[15] Hou J, Brouwer WP, Kreefft K, et al. Unique intrahepatic transcriptomics profiles discriminate the clinical phases of a chronic HBV infection. PLoS ONE. 2017;12(6):e0179920. 28662087 10.1371/journal.pone.0179920 PMC5491066

[16] Wiesweg M, Mairinger F, Reis H, et al. Machine learning reveals a PD-L1-independent prediction of response to immunotherapy of non-small cell lung cancer by gene expression context. Eur J Cancer. 2020;140:76–85. 33059196 10.1016/j.ejca.2020.09.015

[17] Bustamante Eduardo M, Popovici V, Imboden S, et al. Characterization of molecular scores and gene expression signatures in primary breast cancer, local recurrences and brain metastases. BMC Cancer. 2019;19(1):549. 31174485 10.1186/s12885-019-5752-8 PMC6556009

[18] Eastel JM, Lam KW, Lee NL, et al. Application of NanoString technologies in companion diagnostic development. Expert Rev Mol Diagn. 2019;19(7):591–8. 31164012 10.1080/14737159.2019.1623672

[19] Nijman RG, Oostenbrink R, Moll HA, et al. A novel framework for phenotyping children with suspected or confirmed infection for future biomarker studies. Front Pediatr. 2021;9:688272. 34395340 10.3389/fped.2021.688272 PMC8356564

[20] McCrindle BW, Rowley AH, Newburger JW, et al. Diagnosis, treatment, and long-term management of Kawasaki disease: a scientific statement for health professionals from the American heart association. Circulation. 2017;135(17):e927–99. 28356445 10.1161/CIR.0000000000000484

[21] Hoggart C, Shimizu C, Galassini R, et al. Identification of novel locus associated with coronary artery aneurysms and validation of loci for susceptibility to Kawasaki disease. Eur J Hum Genet. 2021;29(12):1734–44. 10.1038/s41431-021-00838-5 33772158 10.1038/s41431-021-00838-5 PMC7994355

[22] Anderson ST, Kaforou M, Brent AJ, et al. Diagnosis of childhood tuberculosis and host RNA expression in Africa. N Engl J Med. 2014;370(18):1712–23. 24785206 10.1056/NEJMoa1303657 PMC4069985

[23] Kampmann B, Seddon JA, Paton J, et al. Evaluating UK national guidance for screening of children for tuberculosis. A prospective multicenter study. Am J Respir Crit Care Med. 2018;197(8):1058–64. 29190430 10.1164/rccm.201707-1487OC PMC5909164

[24] Kuiper R, Wright VJ, Habgood-Coote D, et al. Bridging a diagnostic Kawasaki disease classifier from a microarray platform to a qRT-PCR assay. Pediatr Res. 2023;93(3):559–69. 35732822 10.1038/s41390-022-02148-y PMC9988687

[25] Tian S, Deng J, Huang W, et al. FAM89A and IFI44L for distinguishing between viral and bacterial infections in children with febrile illness. Pediatr Investig. 2021;5(3):195–202. 34589675 10.1002/ped4.12295 PMC8458721

[26] Gomez-Carballa A, Cebey-Lopez M, Pardo-Seco J, et al. A qPCR expression assay of IFI44L gene differentiates viral from bacterial infections in febrile children. Sci Rep. 2019;9(1):11780. 31409879 10.1038/s41598-019-48162-9 PMC6692396

[27] Pennisi I, Rodriguez-Manzano J, Moniri A, et al. Translation of a host blood RNA signature distinguishing bacterial from viral infection into a platform suitable for development as a point-of-care test. JAMA Pediatr. 2021;175(4):417–9. 33393977 10.1001/jamapediatrics.2020.5227 PMC7783591

[28] Roe JK, Thomas N, Gil E, et al. Blood transcriptomic diagnosis of pulmonary and extrapulmonary tuberculosis. JCI Insight. 2016;1(16):e87238. 27734027 10.1172/jci.insight.87238 PMC5053151

[29] Turner CT, Gupta RK, Tsaliki E, et al. Blood transcriptional biomarkers for active pulmonary tuberculosis in a high-burden setting: a prospective, observational, diagnostic accuracy study. Lancet Respir Med. 2020;8(4):407–19. 32178775 10.1016/S2213-2600(19)30469-2 PMC7113842

[30] Gupta RK, Turner CT, Venturini C, et al. Concise whole blood transcriptional signatures for incipient tuberculosis: a systematic review and patient-level pooled meta-analysis. Lancet Respir Med. 2020;8(4):395–406. 31958400 10.1016/S2213-2600(19)30282-6 PMC7113839

[31] Rajan JV, Semitala FC, Mehta T, et al. A Novel, 5-transcript, whole-blood gene-expression signature for tuberculosis screening among people living with human immunodeficiency virus. Clin Infect Dis. 2019;69(1):77–83. 30462176 10.1093/cid/ciy835 PMC6579960

[32] Gliddon HD, Kaforou M, Alikian M, et al. Identification of reduced host transcriptomic signatures for tuberculosis disease and digital PCR-based validation and quantification. Front Immunol. 2021;12:637164. 33763081 10.3389/fimmu.2021.637164 PMC7982854

[33] Martin FJ, Amode MR, Aneja A, et al. Ensembl 2023. Nucleic Acids Res. 2023;51(D1):D933–41. 36318249 10.1093/nar/gkac958 PMC9825606

[34] R Core Team. R: A language and environment for statistical computing. R Foundation for Statistical Computing, Vienna, Austria. 2022. https://www.R-project.org/

[35] DeLong ER, DeLong DM, Clarke-Pearson DL. Comparing the areas under two or more correlated receiver operating characteristic curves: a nonparametric approach. Biometrics. 1988;44(3):837–45. 3203132 10.2307/2531595

[36] Robin X, Turck N, Hainard A, et al. pROC: an open-source package for R and S+ to analyze and compare ROC curves. BMC Bioinformatics. 2011;12:77. 21414208 10.1186/1471-2105-12-77 PMC3068975

[37] Youden WJ. Index for rating diagnostic tests. Cancer. 1950;3(1):32–5. 15405679 10.1002/1097-0142(1950)3:1<32::AID-CNCR2820030106>3.0.CO;2-3

[38] Stevenson M, Sergeant E. epiR: tools for the analysis of epidemiological data. R Package. 2022;2:19.

[39] Kaforou M, Wright VJ, Oni T, et al. Detection of tuberculosis in HIV-infected and -uninfected African adults using whole blood RNA expression signatures: a case-control study. PLoS Med. 2013;10(10):e1001538. 24167453 10.1371/journal.pmed.1001538 PMC3805485

[40] Zou H, Hastie T. Regularization and variable selection via the elastic net. J R Stat Soc Ser B Stat Methodol. 2005;67(2):301–20. 10.1111/j.1467-9868.2005.00503.x

[41] Friedman J, Hastie T, Tibshirani R. Regularization paths for generalized linear models via coordinate descent. J Stat Softw. 2010;33(1):1–22. 20808728 10.18637/jss.v033.i01 PMC2929880

[42] di Iulio J, Bartha I, Spreafico R, Virgin HW, Telenti A. Transfer transcriptomic signatures for infectious diseases. Proc Natl Acad Sci U S A. 2021. 10.1073/pnas.2022486118. 34031243 10.1073/pnas.2022486118 PMC8179160

[43] Hoang LT, Jain P, Pillay TD, et al. Transcriptomic signatures for diagnosing tuberculosis in clinical practice: a prospective, multicentre cohort study. Lancet Infect Dis. 2021;21(3):366–75. 33508221 10.1016/S1473-3099(20)30928-2 PMC7907671

[44] Brogan PA, Bose A, Burgner D, et al. Kawasaki disease: an evidence based approach to diagnosis, treatment, and proposals for future research. Arch Dis Child. 2002;86(4):286–90. 11919108 10.1136/adc.86.4.286 PMC1719139

[45] Bodkin N, Ross M, McClain MT, et al. Systematic comparison of published host gene expression signatures for bacterial/viral discrimination. Genome Med. 2022;14(1):18. 35184750 10.1186/s13073-022-01025-x PMC8858657

[46] Li HK, Kaforou M, Rodriguez-Manzano J, et al. Discovery and validation of a three-gene signature to distinguish COVID-19 and other viral infections in emergency infectious disease presentations: a case-control and observational cohort study. Lancet Microbe. 2021;2(11):e594–603. 34423323 10.1016/S2666-5247(21)00145-2 PMC8367196

[47] Tsalik EL, Fiorino C, Aqeel A, et al. The Host response to viral infections reveals common and virus-specific signatures in the peripheral blood. Front Immunol. 2021;12:741837. 34777354 10.3389/fimmu.2021.741837 PMC8578928

[48] Hamada Y, Penn-Nicholson A, Krishnan S, et al. Are mRNA based transcriptomic signatures ready for diagnosing tuberculosis in the clinic?—A review of evidence and the technological landscape. EBioMedicine. 2022;82:104174. 35850011 10.1016/j.ebiom.2022.104174 PMC9294474

[49] World Health Organization. High priority target product profiles for new tuberculosis diagnostics: report of a consensus meeting. In: Programme GT. 2014. https://www.who.int/publications/i/item/WHO-HTM-TB-2014.18

[50] Irwin AD, Grant A, Williams R, et al. Predicting risk of serious bacterial infections in febrile children in the emergency department. Pediatrics. 2017. 10.1542/peds.2016-2853. 28679639 10.1542/peds.2016-2853

