# Supplementary material for: Integration and validation of host transcript signatures, including a novel 3-transcript tuberculosis signature, to enable one-step multiclass diagnosis of childhood febrile disease
Source: J Transl Med. 2024 Aug 29;22:802. doi: 10.1186/s12967-024-05241-4 (PMC11360490; doi:10.1186/s12967-024-05241-4)
Supplement: Supplementary file 1 — Additional file 1: Supplementary methods and results. [file 12967_2024_5241_MOESM1_ESM.docx]

SUPPLEMENTARY METHODS AND RESULTS

Contents

[Supplementary methods 2](#_Toc164890635)

[Study populations 2](#_Toc164890636)

[TB signature discovery 3](#_Toc164890637)

[Quality control 3](#_Toc164890638)

[Multiclass prediction models 4](#_Toc164890639)

[Supplementary figures 6](#_Toc164890640)

[Figure S1 6](#_Toc164890641)

[Figure S2 7](#_Toc164890642)

[Figure S3 8](#_Toc164890643)

[Figure S4 9](#_Toc164890644)

[Supplementary Tables 10](#_Toc164890645)

[Table S1 10](#_Toc164890646)

[Table S2 13](#_Toc164890647)

[Table S3 14](#_Toc164890648)

[Table S4 15](#_Toc164890649)

[Table S5 19](#_Toc164890650)

[Table S6 20](#_Toc164890651)

[References 21](#_Toc164890652)

# Supplementary methods

## Study populations

GENDRES network (Santiago de Compostela) cohort: Includes 14 patients with viral infections, recruited from hospitals within the GENDRES network in Spain, between March 2010 and February 2015. Patients were included if they had a fever (axillary temperature ≥38°C) & a clinical indication for blood testing. All patients underwent routine investigations as part of clinical care, including complete blood cell count and differential, C-reactive protein (CRP) level, blood chemistries, blood and urine cultures, and cerebrospinal fluid analysis where indicated. Clinical group assignment in the original study was made after review of investigation results using previously agreed definitions, ^1^ by independent consensus of 2 clinicians. Specifically, the definite viral infection group included only patients with culture, molecular, or immunofluorescent test–confirmed viral infection and no features of coexisting bacterial infection, and with CRP and neutrophil cut-offs of CRP ≤60 mg/L and neutrophils ≤12,000/μL. Blood samples were collected as soon as possible after presentation, and before clinical diagnosis was confirmed.

ILULU consortium cohort: Includes 16 patients with pulmonary tuberculosis, recruited from The Red Cross War Memorial Children’s Hospital, Cape Town, between March 2010 and June 2011. ^2^ Children younger than 15 years of age were recruited into the original study if they had one of: cough; fever; weight loss of more than 2 weeks’ duration; pneumonia that was unresponsive to antibiotics; any other clinical findings that were suggestive of tuberculosis; or a history of close contact with an adult with tuberculosis. All patients underwent a panel of investigations, including chest radiography, blood-testing, a serologic test or polymerase chain-reaction (PCR) assay for HIV, and a tuberculin skin test, with or without an Interferon-gamma release assay (IGRA). Induced sputum samples and a specimen of tissue or cerebrospinal fluid (if clinically indicated) were examined for acid-fast bacilli and cultured for mycobacteria. Culture-confirmed tuberculosis was defined as the isolation of M. tuberculosis from a child with clinical features of tuberculosis. All 16 CT TB samples were sputum-positive for M. tuberculosis, and were HIV-negative. PAXgene collection was prior to initiation of treatment. One patient had previously received antituberculosis treatment ending one year prior to inclusion, and had sputum-positive TB when enrolled into this study.

EUCLIDS consortium cohort: Includes patients with a variety of diseases: 24 bacterial infections; 6 viral infections; 3 with Kawasaki disease; & 16 controls. Patients were recruited across multiple European hospitals between March 2010 and November 2015. Eligibility criteria included age between 1-month and 18-years, and admission to hospital with sepsis (or suspected sepsis) or severe focal infection, or both. Patients were enrolled based on their clinical characteristics at presentation, typically before confirmatory microbiology test results were available. All patients underwent routine investigations and microbiological diagnosis as part of clinical care using locally available diagnostic procedures as appropriate. Clinical group assignment in the original study was made after review of investigation results using previously agreed definitions, ^3^ with phenotyping according to the likelihood of bacterial infection. CRP and absolute neutrophil count cut-offs were used to characterise the definite viral infection phenotype.

UK Kawasaki Genetics Consortium cohort: We included a total of 15 patients recruited after presenting to Paediatric centres in the UK between June 2014 and December 2016 with a diagnosis of presumed Kawasaki Disease. Patients underwent routine clinical assessment, including echocardiogram and assessment for coronary artery aneurysms. Clinical assignment was made according to the American Heart Association KD diagnostic criteria into categories of complete, incomplete, or unlikely KD. ^4^ Patients with unlikely KD were excluded from downstream analysis.

NIKS TB: We included 2 patients from the NIKS TB study. ^5^ Patients were recruited in the UK between 2011-2014 from outpatient clinics, as part of an observational study into paediatric TB. Children were diagnosed with TB based on positive identification of *M. tuberculosis*, or if they had clinical features of TB together with a positive Mantoux or interferon gamma release assay.

## TB signature discovery

The microarray discovery and validation datasets described by Anderson et al. ^2^ were separately pre-processed and normalised using the lumi R package. ^6^ The mean raw intensity values were corrected for local background intensities, variance stabilised, and a robust spline normalisation was applied to each array. ^7^ The discovery dataset was randomly split into a training (223 samples: 88 microbiologically confirmed TB and 135 unlikely TB) and a test set (57 samples: 23 microbiologically confirmed TB and 34 unlikely TB) for signature discovery. Differential expression analysis accounting for age and sex was performed on the training set using the limma R package. ^8^ 207 genes were significantly differentially expressed (SDE) (199 genes over-expressed and 8 under-expressed in TB), i.e., log_2_FoldChange <-0.5 or >0.5 and Benjamini-Hochberg procedure adjusted p-value <0.01, between the two disease groups of interest in the training set. SDE genes were taken forward to feature selection with FS-PLS. ^1,9^ A 3-transcript signature (*GBP6*, *KIFC3* and *CYB561*) was identified as the best combination of genes for discriminating between microbiologically confirmed TB and unlikely TB. A disease risk score (DRS) was calculated for each patient in the training, test and validation sets and the performance of the signature was evaluated in terms of AUC, sensitivity, and specificity (Table S5). ^2^

Boxplots of the DRS and ROC curves of the 3-transcript signature in the training, test and validation sets are shown in Figure 4 of the main paper.

## Quality control

Technical quality control (QC) on raw data was performed as recommended by NanoString, ^10^ including assessment for: imaging quality; binding density; positive control linearity; and limit of detection. Binding density values were on the lower range of acceptability (0.1–2.25), with only one sample marginally below the lower limit. This sample did not fail any remaining QC protocols and was therefore included in final analysis.

Data normalisation was undertaken using the following sequential workflow:

1. Positive control (PC) normalisation: For each sample we calculated the geometric mean of 5 PC probes, spiked into each assay. As suggested by the manufacturers we excluded the PC probe POS_F as this is present at concentrations near the limit of detection of the assay. The mean of these sample-specific geometric means was then divided by each geometric mean to obtain a sample-specific PC normalisation factor. We then multiplied all counts for each sample by the sample-specific PC normalisation factor.
2. Background subtraction: An estimate of background non-specific binding was obtained by taking the median of 8 negative control (NC) probes for each sample. A liberal approach was taken by using the median as an estimate to avoid over subtraction of noise. This sample-specific background level was subtracted from each sample, and levels below a corrected count of 1 were recalibrated to 1.
3. Housekeeping normalisation: Using the 7-housekeeping transcripts which passed our quality control assessment (see below) we calculated housekeeping normalisation factors in an identical fashion to the above method for PC normalisation factors, and calculated final normalised counts by multiplying by these factors. Any counts that were previously recalibrated from below the count level of 1 from step two were reset to 1.

Additional QC was performed on raw data and normalised data as described below:

- Raw counts and variation in housekeeping transcripts across samples: The housekeeping transcript JTB was excluded based on assessment of raw counts, which demonstrated extremely low counts across all samples. Additionally, housekeeping transcript HBB demonstrated very high variability relative to expression levels and was also excluded. Notably, removal of these transcripts is also equivalent to minimising the pairwise variation statistic, as recommended by NanoString.
- Raw counts and limit of detection per-sample: Inspection of raw counts relative to the limit of detection revealed two samples with the majority of both housekeeping and endogenous transcripts detected at levels below the limit of detection. These samples were retained for further QC assessment.
- Positive and Housekeeping normalisation factors: Positive control normalisation factors were all within a narrow range from 0.7-1.7, all within the suggested 3-fold QC threshold. This suggests minimal technical variability (independent of sample input variability). Two samples had housekeeping normalisation factors outside of the suggested QC thresholds of 0.1–10 (10.9 & 22.3 respectively). Although the first of these is only marginally above the QC threshold, as both samples also flagged for low overall expression across transcripts, we have removed these samples from subsequent analysis.
- Principal component analysis (PCA), pre- and post-normalisation: Pre-normalisation PCA demonstrated the 1^st^ principal component (PC1) explained over 44% of the variation and was dominated by the effect of the total sum of expression across each sample (Figure S4, correlation between PC1 and expression-sum = 0.84). There is also a single outlier sample pre-normalisation in PC2. Post-normalisation we observed a reduction in the effect from expression-sum on PC1 (PC1 explained 29% of the variation, and PC1 – expression sum correlation = 0.59). There are no outlier samples. The importance of disease effect also increased in PC1 and lower order principal components. Given the high expression levels and variability across disease groups of specific transcripts in our codeset, such as the interferon-stimulated genes, we do not expect the expression sum to be uncorrelated with our higher order principal components.

## Multiclass prediction models

We fit two separate models to predict the diagnostic class of each of the 76 non-control samples out of the four options – DB, DV, KD or TB. For both models control samples were removed for model training, as healthy children are not the target group for such a diagnostic test. We then restricted the transcript list of interest by performing a Mann-Whitney-U (MWU) test for each transcript on one-vs-all comparisons based on the previously discovered diagnostic comparisons reported in Table S1. For example, RIPK2 is listed for the comparison KD vs other febrile conditions, and hence the MWU test was performed on the comparison KD vs other diseases. Transcripts with comparison listed as Bacterial vs Viral had two p-values calculated, one for each comparison (DB vs other, DV vs other). We then corrected p-values for multiple testing using the method of Benjamini & Hochberg. ^11^ Transcripts with a p-value <0.1 were taken forward to model derivation.

Mixed One-vs-All model (MOVA-model):

Our MOVA-model works by fitting 4 separate models and combining the resulting probability predictions. For each one-vs-all comparison (DB vs other; DV vs other; KD vs other; TB vs other) we fit a relaxed, regularised binomial logistic regression model, implemented in glmnet, ^12^ with alpha parameter in the elastic net set to 0.95 to simulate a quasi-LASSO model. The models were trained using 10-fold cross validation, and lambda value of lambda.1se was used to determine the final models. Samples were weighted according to the size of their diagnostic classes in each comparison, in order to account for unbalanced case numbers. The final predicted disease for each sample was the disease with the highest probability from the four separate models.

Multiclass model:

To avoid the theoretical problems associated with running multiple diagnostic tests in-parallel we then fit a single multiclass prediction model, using a relaxed, regularised multinomial logistic regression model, implemented in glmnet, with an otherwise identical workflow to the models described above. For each patient the model output provides prediction probabilities for each of the four diagnostic categories. Unlike the MOVA-model these 4 patient-specific probabilities all sum to 1.

# Supplementary figures

## Figure S1

*Figure S1: Wright13 signature DRS across diseases, separated by day of illness from symptom onset (for KD patients this is from fever onset). DRS, Disease Risk Score.*


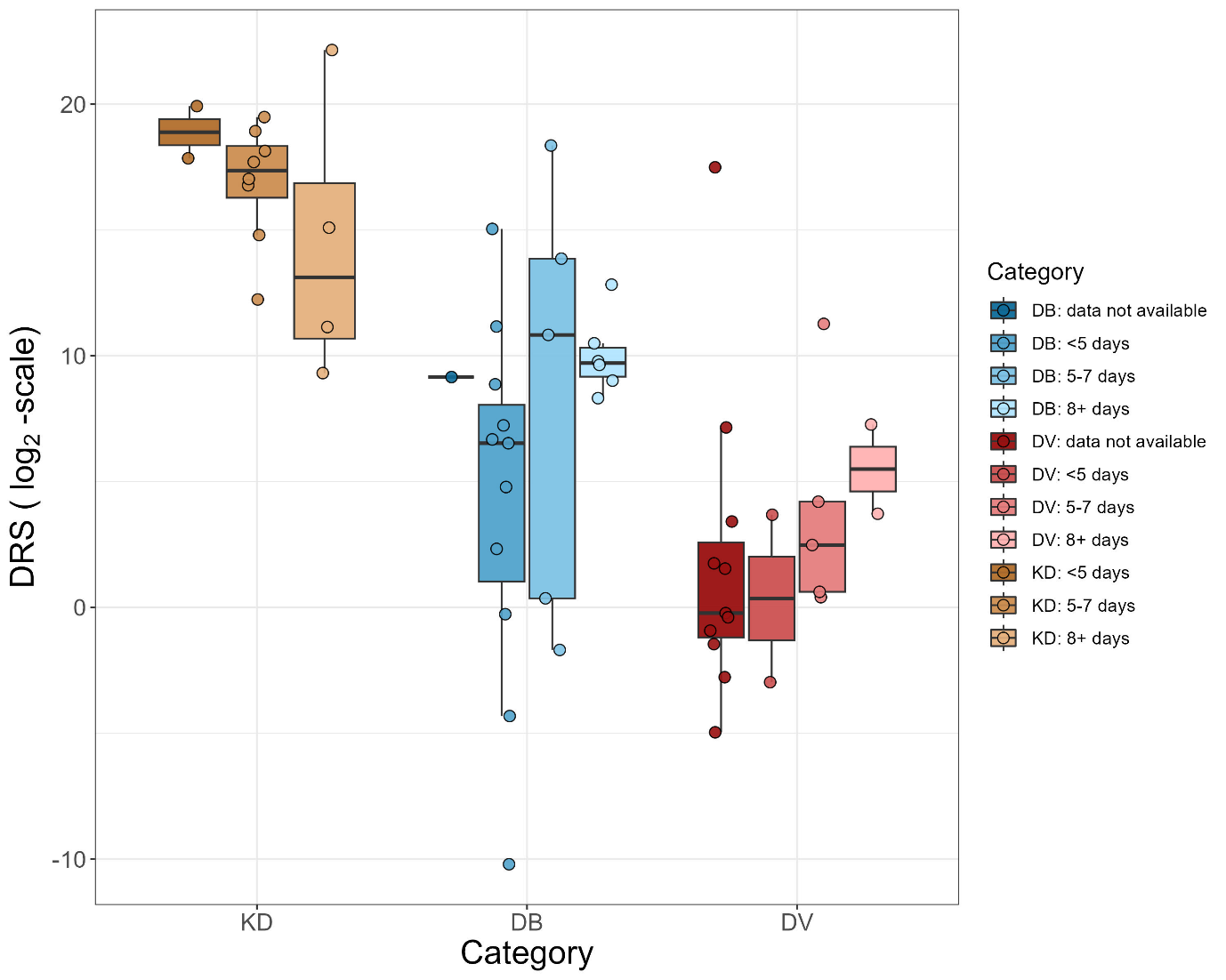


## Figure S2


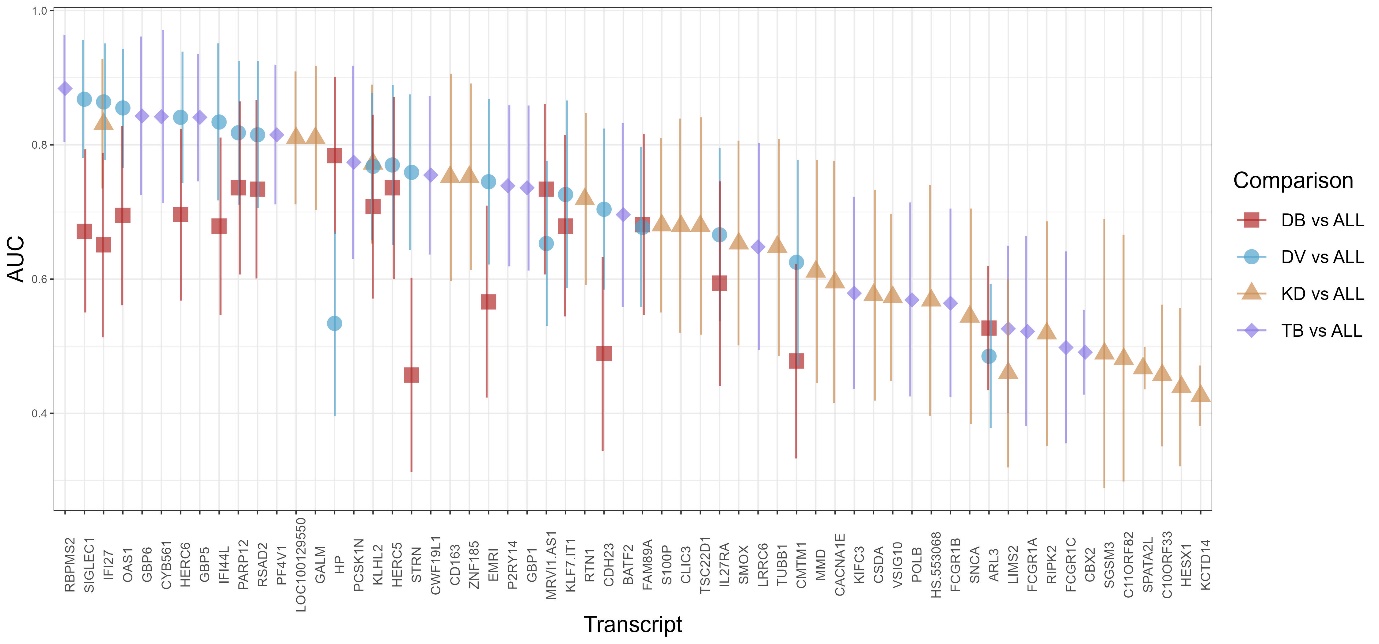


*Figure S2: AUCs for transcript-specific one-vs-all comparisons. Transcript AUCs are only included if meeting the significance threshold described in the methods section. Transcripts on the x-axis ordered by maximum AUC across the transcript-specific comparisons. Solid vertical lines represent 95% confidence intervals for each AUC, coloured according to the disease compared. AUC, Area Under ROC* Curve.

## Figure S3

*Figure S3: Overview of MOVA-model transcript selection.*


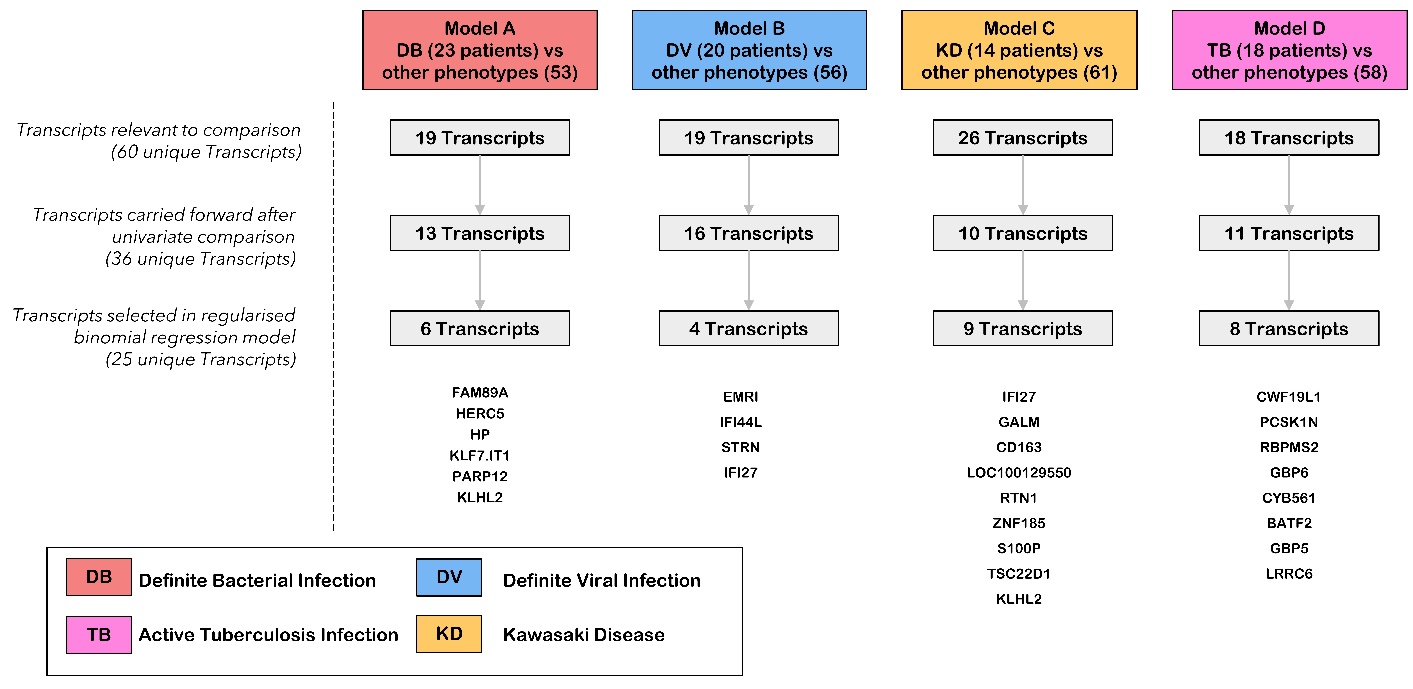


## Figure S4


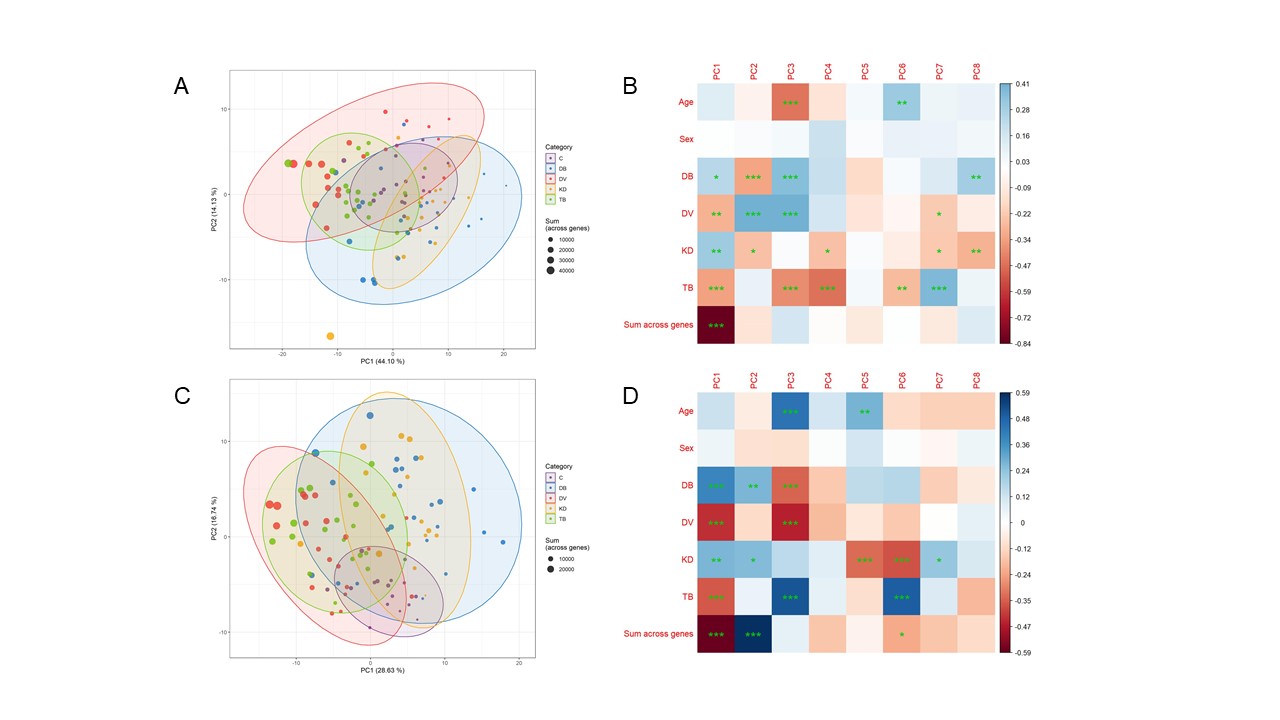


*Figure S4: Principal component analyses pre- and post-normalisation. A) PC1 vs PC2 pre-normalisation. Samples coloured by disease, with 95% confidence ellipses plotted per-disease group, and point size proportional to expression-sum across all transcripts per-sample. B) Correlation of first 8 principal components with clinical features pre-normalisation, Binary variables treated as 1 – 0 numeric variables. C) PC1 vs PC2 post-normalisation. D) Correlation of first 8 principal components with clinical features post-normalisation. P-values for correlations: * <0.05, ** <0.01, *** <0.001*

# Supplementary Tables

## Table S1

*Table S1: transcript selections. SDE, Significantly differentially expressed*

| Transcript ID | Comparisons | Validation Signature | Discovery platform | Reason for  inclusion |
| --- | --- | --- | --- | --- |
| BATF2 | TB vs other diseases; TB vs healthy control | BATF2^13^ | Microarray | Existing signature |
| FAM89A | Bacterial vs Viral | Herberg2^1^ | Microarray | Existing signature |
| IFI44L | Bacterial vs Viral | Herberg2/ Pennisi2 | Microarray | Existing signature |
| GBP6 | TB vs other diseases | TB3^2^ | Microarray | Existing signature |
| KIFC3 | TB vs other diseases | TB3 | Microarray | Existing signature |
| CYB561 | TB vs other diseases; TB vs Bacterial or Viral infection | TB3 | Microarray | Existing signature |
| IFI27 | Bacterial vs Viral; KD vs other febrile conditions | Wright13^14^ | Microarray | Existing signature |
| KLHL2 | Bacterial vs Viral; KD vs other febrile conditions | Wright13 | Microarray | Existing signature |
| C10ORF33 | KD vs other febrile conditions | Wright13 | Microarray | Existing signature |
| C11ORF82 | KD vs other febrile conditions | Wright13 | Microarray | Existing signature |
| CACNA1E | KD vs other febrile conditions | Wright13 | Microarray | Existing signature |
| CD163 | KD vs other febrile conditions | Wright13 | Microarray | Existing signature |
| CLIC3 | KD vs other febrile conditions | Wright13 | Microarray | Existing signature |
| HS.553068 | KD vs other febrile conditions | Wright13 | Microarray | Existing signature |
| LOC100129550 | KD vs other febrile conditions | Wright13 | Microarray | Existing signature |
| RTN1 | KD vs other febrile conditions | Wright13 | Microarray | Existing signature |
| S100P | KD vs other febrile conditions | Wright13 | Microarray | Existing signature |
| SMOX | KD vs other febrile conditions | Wright13 | Microarray | Existing signature |
| ZNF185 | KD vs other febrile conditions | Wright13 | Microarray | Existing signature |
| EMRI | Bacterial vs Viral | Pennisi2^15^ | Microarray | Existing signature |
| ARL3 | Bacterial vs Viral |  | RNAseq | SDE in the Coote et al dataset |
| CDH23 | Bacterial vs Viral |  | Microarray | SDE in the Herberg et al dataset |
| CMTM1 | Bacterial vs Viral |  | Microarray | SDE in the Herberg et al dataset |
| HERC5 | Bacterial vs Viral |  | RNAseq | SDE in the Coote et al dataset |
| HERC6 | Bacterial vs Viral |  | RNAseq | SDE in the Coote et al dataset |
| HP | Bacterial vs Viral |  | RNAseq | SDE in the Coote et al dataset |
| IL27RA | Bacterial vs Viral |  | RNAseq | SDE in the Coote et al dataset |
| KLF7-IT1 | Bacterial vs Viral |  | RNAseq | SDE in the Coote et al dataset |
| MIR3128 | Bacterial vs Viral |  | RNAseq | SDE in the Coote et al dataset |
| MRVI1-AS1 | Bacterial vs Viral |  | RNAseq | SDE in the Coote et al dataset |
| OAS1 | Bacterial vs Viral |  | Microarray | SDE in the Herberg et al dataset |
| PARP12 | Bacterial vs Viral |  | RNAseq | SDE in the Coote et al dataset |
| RSAD2 | Bacterial vs Viral |  | Microarray | SDE in the Herberg et al dataset |
| SIGLEC1 | Bacterial vs Viral |  | RNAseq | SDE in the Coote et al dataset |
| STRN | Bacterial vs Viral |  | RNAseq | SDE in the Coote et al dataset |
| CSDA | KD vs Bacterial or Viral infection |  | Microarray | SDE in the Wright et al dataset |
| GALM | KD vs Bacterial or Viral infection |  | Microarray | SDE in the Wright et al dataset |
| HESX1 | KD vs Bacterial or Viral infection |  | Microarray | SDE in the Wright et al dataset |
| KCTD14 | KD vs Bacterial or Viral infection |  | Microarray | SDE in the Wright et al dataset |
| MMD | KD vs Bacterial or Viral infection |  | Microarray | SDE in the Wright et al dataset |
| SNCA | KD vs Bacterial or Viral infection |  | Microarray | SDE in the Wright et al dataset |
| TSC22D1 | KD vs Bacterial or Viral infection |  | Microarray | SDE in the Wright et al dataset |
| TUBB1 | KD vs Bacterial or Viral infection |  | Microarray | SDE in the Wright et al dataset |
| RIPK2 | KD vs other febrile conditions |  | RNAseq | SDE in the Coote et al dataset |
| SGSM3 | KD vs other febrile conditions |  | RNAseq | SDE in the Coote et al dataset |
| SPATA2L | KD vs other febrile conditions |  | RNAseq | SDE in the Coote et al dataset |
| VSIG10 | KD vs other febrile conditions |  | RNAseq | SDE in the Coote et al dataset |
| CBX2 | TB vs Bacterial or Viral infection |  | Microarray | SDE in the Anderson et al dataset |
| CWF19L1 | TB vs Bacterial or Viral infection |  | Microarray | SDE in the Anderson et al dataset |
| LRRC6 | TB vs Bacterial or Viral infection |  | Microarray | SDE in the Anderson et al dataset |
| PCSK1N | TB vs Bacterial or Viral infection |  | Microarray | SDE in the Anderson et al dataset |
| PF4V1 | TB vs Bacterial or Viral infection |  | Microarray | SDE in the Anderson et al dataset |
| RBPMS2 | TB vs Bacterial or Viral infection |  | Microarray | SDE in the Anderson et al dataset |
| LIMS2 | TB vs Bacterial or Viral infection; KD vs other febrile conditions |  | Microarray | SDE in the Anderson et al dataset |
| FCGR1B | TB vs healthy control |  | Microarray | SDE in the Anderson et al dataset |
| FCGR1C | TB vs healthy control |  | Microarray | SDE in the Anderson et al dataset |
| FCGR1A | TB vs other diseases |  | Microarray | SDE in the Anderson et al dataset |
| GBP1 | TB vs other diseases |  | Microarray | SDE in the Anderson et al dataset |
| GBP5 | TB vs other diseases |  | Microarray | SDE in the Anderson et al dataset |
| P2RY14 | TB vs other diseases |  | Microarray | SDE in the Anderson et al dataset |
| POLB | TB vs other diseases |  | Microarray | SDE in the Anderson et al dataset |

## Table S2

*Table S2: Organisms detected in DB and DV patients. Rhinovirus and/or RSV found as co-infection in one DV patient, and as additional organisms in 2 separate DB cases. RSV, Respiratory syncytial virus.*

|  | Organism | N |
| --- | --- | --- |
| Bacteria | Coliform, unspecified | 1 |
|  | Escherichia coli | 3 |
|  | Kingella spp. | 2 |
|  | Neisseria meningitidis | 3 |
|  | Salmonella spp. | 2 |
|  | Streptococcus, group A | 1 |
|  | Pneumococcus spp. | 8 |
|  | Staphylococcus aureus | 3 |
| Viruses | Adenovirus | 1 |
|  | Ebstein Barr Virus | 1 |
|  | Enterovirus | 2 |
|  | Parechovirus | 1 |
|  | Rhinovirus* | 3 |
|  | Rotavirus | 3 |
|  | RSV* | 12 |

## Table S3

*Table S3: Area under receiver operator characteristic curve (AUC) for C-reactive protein (CRP), White blood cells (WBC), neutrophils and lymphocytes for between-group and one-vs-all disease comparisons.* CRP and neutrophil count cutoffs were used for phenotyping definite viral (DV) patients. **CRP values only available for two TB patients.*

| Comparison | CRP | WBC | Neutrophils | Lymphocytes |
| --- | --- | --- | --- | --- |
| DB vs DV | 0.800 [0.628 - 0.972]* | 0.518 [0.328 - 0.708] | 0.669 [0.480 - 0.857]* | 0.682 [0.494 - 0.870] |
| DB vs KD | 0.512 [0.310 - 0.713] | 0.606 [0.418 - 0.794] | 0.578 [0.378 - 0.779] | 0.473 [0.278 - 0.668] |
| DB vs TB | 0.850 [0.689 - 1.000] ]** | 0.491 [0.302 - 0.680] | 0.608 [0.414 - 0.802] | 0.630 [0.439 - 0.821] |
| DV vs KD | 0.923 [0.790 - 1.000]* | 0.750 [0.564 - 0.936] | 0.932 [0.839 - 1.000]* | 0.779 [0.590 - 0.968] |
| DV vs TB | 0.821 [0.536 - 1.000] ]*^,^ ** | 0.482 [0.254 - 0.710] | 0.559 [0.314 - 0.805]* | 0.566 [0.322 - 0.810] |
| KD vs TB | 0.962 [0.855 - 1.000]** | 0.645 [0.420 - 0.871] | 0.808 [0.633 - 0.982] | 0.703 [0.499 - 0.907] |
| DB vs other | 0.620 [0.443 - 0.798]* | 0.533 [0.363 - 0.703] | 0.557 [0.380 - 0.734]* | 0.587 [0.422 - 0.752] |
| DV vs other | 0.810 [0.678 - 0.942]* | 0.581 [0.435 - 0.727] | 0.716 [0.583 - 0.849]* | 0.679 [0.522 - 0.836] |
| KD vs other | 0.642 [0.472 - 0.812]* | 0.654 [0.524 - 0.784] | 0.731 [0.608 - 0.854]* | 0.640 [0.485 - 0.794] |
| TB vs other | 0.881 [0.778 - 0.984]*^,^ ** | 0.532 [0.350 - 0.714] | 0.629 [0.458 - 0.799]* | 0.605 [0.432 - 0.778] |

## Table S4

*Table S4: Expression values and performance for one-vs-all comparisons for each transcript, based on original study comparisons (supplementary table, transcript selections). Transcripts ranked by AUC. AUC, Area Under ROC Curve; BH, Benjamini & Hochberg method; MWU, Mann-Whitney-U.*

|  |  |  |  |  |  | Median Expression, by group | | | |
| --- | --- | --- | --- | --- | --- | --- | --- | --- | --- |
| Transcript | Comparison | Log2-Fold-Change | AUC | P-value MWU Test | P-value MWU Test, BH-Corrected | DB | DV | KD | TB |
| RBPMS2 | TB vs ALL | 4.31 | 0.884 [0.804 - 0.964] | 5.75E-07 | 1.95E-05 | 2.7 | 1.0 | 1.0 | 19.9 |
| SIGLEC1 | DV vs ALL | 6.05 | 0.868 [0.780 - 0.956] | 2.39E-07 | 1.95E-05 | 1.0 | 66.2 | 1.0 | 5.4 |
| IFI27 | DV vs ALL | 6.04 | 0.864 [0.777 - 0.951] | 1.30E-06 | 2.67E-05 | 1.1 | 351.8 | 1.0 | 31.6 |
| OAS1 | DV vs ALL | 2.69 | 0.855 [0.766 - 0.943] | 3.06E-06 | 4.63E-05 | 172.4 | 1501.9 | 168.7 | 460.6 |
| GBP6 | TB vs ALL | 3.09 | 0.843 [0.725 - 0.961] | 8.42E-06 | 8.63E-05 | 1.0 | 3.3 | 5.0 | 16.2 |
| CYB561 | TB vs ALL | 2.44 | 0.842 [0.713 - 0.971] | 7.15E-07 | 1.95E-05 | 1.0 | 1.0 | 1.0 | 5.4 |
| GBP5 | TB vs ALL | 2.34 | 0.841 [0.746 - 0.936] | 1.45E-05 | 1.19E-04 | 72.4 | 192.7 | 177.5 | 613.2 |
| HERC6 | DV vs ALL | 1.88 | 0.841 [0.743 - 0.939] | 7.19E-06 | 8.42E-05 | 15.4 | 73.1 | 11.0 | 35.6 |
| IFI44L | DV vs ALL | 3.98 | 0.834 [0.717 - 0.951] | 1.08E-05 | 9.84E-05 | 50.0 | 1412.6 | 30.7 | 254.8 |
| IFI27 | KD vs ALL | -5.24 | 0.831 [0.735 - 0.928] | 1.03E-04 | 5.63E-04 | 1.1 | 351.8 | 1.0 | 31.6 |
| PARP12 | DV vs ALL | 1.57 | 0.818 [0.711 - 0.925] | 2.82E-05 | 2.10E-04 | 44.7 | 157.4 | 46.1 | 118.0 |
| PF4V1 | TB vs ALL | 2.2 | 0.815 [0.711 - 0.919] | 6.31E-05 | 3.98E-04 | 10.4 | 24.4 | 46.8 | 93.5 |
| RSAD2 | DV vs ALL | 2.85 | 0.815 [0.706 - 0.925] | 3.30E-05 | 2.26E-04 | 61.7 | 948.6 | 60.6 | 398.4 |
| LOC100129550 | KD vs ALL | 1.08 | 0.810 [0.711 - 0.909] | 3.23E-04 | 1.57E-03 | 33.8 | 17.2 | 51.2 | 21.3 |
| GALM | KD vs ALL | -2.01 | 0.810 [0.703 - 0.917] | 3.26E-04 | 1.57E-03 | 10.3 | 30.6 | 5.8 | 26.8 |
| HP | DB vs ALL | 2.99 | 0.784 [0.667 - 0.901] | 9.63E-05 | 5.63E-04 | 300.2 | 63.1 | 89.3 | 5.8 |
| PCSK1N | TB vs ALL | 1.16 | 0.774 [0.630 - 0.918] | 3.39E-06 | 4.63E-05 | 1.0 | 1.0 | 1.0 | 2.2 |
| KLHL2 | KD vs ALL | 1.37 | 0.772 [0.653 - 0.890] | 1.65E-03 | 5.01E-03 | 209.2 | 72.6 | 237.1 | 81.9 |
| HERC5 | DV vs ALL | 1.53 | 0.770 [0.651 - 0.889] | 3.82E-04 | 1.74E-03 | 39.2 | 288.1 | 51.6 | 145.7 |
| KLHL2 | DV vs ALL | -1.3 | 0.768 [0.658 - 0.878] | 4.18E-04 | 1.80E-03 | 209.2 | 72.6 | 237.1 | 81.9 |
| STRN | DV vs ALL | -0.44 | 0.759 [0.643 - 0.875] | 6.53E-04 | 2.68E-03 | 132.7 | 111.4 | 160.2 | 161.6 |
| CWF19L1 | TB vs ALL | -0.16 | 0.755 [0.637 - 0.873] | 1.18E-03 | 4.07E-03 | 82.8 | 74.0 | 77.7 | 69.8 |
| ZNF185 | KD vs ALL | 1.2 | 0.752 [0.613 - 0.891] | 3.54E-03 | 9.07E-03 | 37.0 | 23.0 | 66.8 | 46.2 |
| CD163 | KD vs ALL | -1.53 | 0.752 [0.597 - 0.906] | 3.53E-03 | 9.07E-03 | 59.1 | 24.7 | 10.1 | 28.9 |
| EMRI | DV vs ALL | -1.31 | 0.745 [0.622 - 0.869] | 1.24E-03 | 4.07E-03 | 111.6 | 48.6 | 108.9 | 122.1 |
| P2RY14 | TB vs ALL | 2.12 | 0.739 [0.619 - 0.859] | 2.36E-03 | 6.91E-03 | 7.2 | 8.3 | 57.0 | 59.1 |
| GBP1 | TB vs ALL | 1.39 | 0.736 [0.613 - 0.859] | 2.74E-03 | 7.75E-03 | 102.8 | 390.9 | 248.4 | 600.5 |
| PARP12 | DB vs ALL | -1.17 | 0.736 [0.607 - 0.865] | 1.22E-03 | 4.07E-03 | 44.7 | 157.4 | 46.1 | 118.0 |
| HERC5 | DB vs ALL | -1.86 | 0.736 [0.600 - 0.871] | 1.22E-03 | 4.07E-03 | 39.2 | 288.1 | 51.6 | 145.7 |
| MRVI1.AS1 | DB vs ALL | 3.48 | 0.734 [0.607 - 0.861] | 9.38E-04 | 3.66E-03 | 19.3 | 1.7 | 11.6 | 1.0 |
| RSAD2 | DB vs ALL | -2.54 | 0.734 [0.601 - 0.867] | 1.32E-03 | 4.16E-03 | 61.7 | 948.6 | 60.6 | 398.4 |
| KLF7.IT1 | DV vs ALL | -0.85 | 0.726 [0.587 - 0.866] | 2.91E-03 | 7.95E-03 | 50.2 | 22.5 | 45.3 | 36.9 |
| RTN1 | KD vs ALL | -2.24 | 0.719 [0.591 - 0.847] | 8.99E-03 | 1.99E-02 | 2.2 | 1.8 | 1.0 | 14.3 |
| KLHL2 | DB vs ALL | 1.2 | 0.708 [0.571 - 0.845] | 4.30E-03 | 1.07E-02 | 209.2 | 72.6 | 237.1 | 81.9 |
| CDH23 | DV vs ALL | -1.57 | 0.704 [0.584 - 0.824] | 4.43E-03 | 1.07E-02 | 1.0 | 1.0 | 2.0 | 3.1 |
| HERC6 | DB vs ALL | -1.17 | 0.696 [0.568 - 0.824] | 7.16E-03 | 1.68E-02 | 15.4 | 73.1 | 11.0 | 35.6 |
| BATF2 | TB vs ALL | 1.55 | 0.696 [0.559 - 0.833] | 1.29E-02 | 2.64E-02 | 18.6 | 38.1 | 41.8 | 83.1 |
| OAS1 | DB vs ALL | -1.43 | 0.695 [0.561 - 0.828] | 7.55E-03 | 1.72E-02 | 172.4 | 1501.9 | 168.7 | 460.6 |
| FAM89A | DB vs ALL | 1.81 | 0.681 [0.546 - 0.816] | 1.12E-02 | 2.42E-02 | 10.8 | 1.9 | 1.0 | 7.4 |
| S100P | KD vs ALL | 1.17 | 0.680 [0.550 - 0.810] | 3.68E-02 | 6.48E-02 | 847.4 | 209.7 | 519.0 | 17.3 |
| IFI44L | DB vs ALL | -2.45 | 0.679 [0.547 - 0.811] | 1.41E-02 | 2.77E-02 | 50.0 | 1412.6 | 30.7 | 254.8 |
| KLF7.IT1 | DB vs ALL | 0.51 | 0.679 [0.544 - 0.814] | 1.42E-02 | 2.77E-02 | 50.2 | 22.5 | 45.3 | 36.9 |
| CLIC3 | KD vs ALL | -1.7 | 0.679 [0.520 - 0.839] | 3.80E-02 | 6.48E-02 | 11.1 | 34.0 | 9.2 | 48.7 |
| TSC22D1 | KD vs ALL | 0.83 | 0.679 [0.517 - 0.841] | 3.81E-02 | 6.48E-02 | 77.4 | 112.8 | 201.8 | 175.4 |
| FAM89A | DV vs ALL | -1.69 | 0.677 [0.558 - 0.797] | 1.73E-02 | 3.30E-02 | 10.8 | 1.9 | 1.0 | 7.4 |
| SIGLEC1 | DB vs ALL | -2.57 | 0.671 [0.550 - 0.793] | 1.23E-02 | 2.59E-02 | 1.0 | 66.2 | 1.0 | 5.4 |
| IL27RA | DV vs ALL | 0.36 | 0.666 [0.537 - 0.795] | 2.88E-02 | 5.37E-02 | 27.3 | 38.3 | 26.2 | 33.9 |
| MRVI1.AS1 | DV vs ALL | -2.05 | 0.653 [0.530 - 0.776] | 3.87E-02 | 6.48E-02 | 19.3 | 1.7 | 11.6 | 1.0 |
| SMOX | KD vs ALL | 1.1 | 0.653 [0.501 - 0.806] | 7.52E-02 | 1.21E-01 | 4.4 | 5.9 | 23.3 | 29.0 |
| IFI27 | DB vs ALL | -5.22 | 0.651 [0.513 - 0.788] | 3.70E-02 | 6.48E-02 | 1.1 | 351.8 | 1.0 | 31.6 |
| LRRC6 | TB vs ALL | 1.03 | 0.648 [0.494 - 0.803] | 6.02E-02 | 9.87E-02 | 94.0 | 77.1 | 117.5 | 204.6 |
| TUBB1 | KD vs ALL | 0.64 | 0.648 [0.486 - 0.809] | 8.79E-02 | 1.39E-01 | 308.8 | 301.5 | 521.0 | 658.6 |
| CMTM1 | DV vs ALL | -0.58 | 0.625 [0.471 - 0.778] | 1.02E-01 | 1.58E-01 | 105.9 | 80.4 | 189.0 | 101.0 |
| MMD | KD vs ALL | 0.52 | 0.611 [0.445 - 0.777] | 1.99E-01 | 2.88E-01 | 48.0 | 67.2 | 97.4 | 123.0 |
| CACNA1E | KD vs ALL | 0.51 | 0.595 [0.415 - 0.776] | 2.69E-01 | 3.80E-01 | 10.6 | 6.5 | 15.1 | 13.1 |
| IL27RA | DB vs ALL | -0.33 | 0.594 [0.441 - 0.746] | 2.00E-01 | 2.88E-01 | 27.3 | 38.3 | 26.2 | 33.9 |
| KIFC3 | TB vs ALL | 1.39 | 0.579 [0.436 - 0.722] | 3.04E-01 | 4.23E-01 | 4.7 | 1.5 | 1.3 | 4.3 |
| CSDA | KD vs ALL | 0.14 | 0.576 [0.419 - 0.733] | 3.80E-01 | 4.89E-01 | 148.6 | 174.1 | 259.2 | 404.1 |
| VSIG10 | KD vs ALL | 0 | 0.573 [0.448 - 0.697] | 1.90E-01 | 2.83E-01 | 1.0 | 1.0 | 1.0 | 1.0 |
| POLB | TB vs ALL | 0.1 | 0.569 [0.425 - 0.714] | 3.82E-01 | 4.89E-01 | 45.3 | 64.4 | 85.2 | 66.4 |
| HS.553068 | KD vs ALL | -0.64 | 0.568 [0.396 - 0.740] | 4.34E-01 | 5.39E-01 | 10.4 | 11.9 | 9.4 | 26.8 |
| EMRI | DB vs ALL | 0.33 | 0.566 [0.423 - 0.709] | 3.67E-01 | 4.85E-01 | 111.6 | 48.6 | 108.9 | 122.1 |
| FCGR1B | TB vs ALL | 0.39 | 0.564 [0.424 - 0.705] | 4.16E-01 | 5.25E-01 | 111.9 | 110.0 | 227.0 | 160.2 |
| SNCA | KD vs ALL | -0.47 | 0.544 [0.384 - 0.705] | 6.10E-01 | 7.05E-01 | 259.0 | 425.8 | 404.4 | 1266.5 |
| HP | DV vs ALL | -0.23 | 0.534 [0.396 - 0.671] | 6.62E-01 | 7.54E-01 | 300.2 | 63.1 | 89.3 | 5.8 |
| ARL3 | DB vs ALL | 0 | 0.527 [0.434 - 0.619] | 5.71E-01 | 6.69E-01 | 1.0 | 1.0 | 1.0 | 1.0 |
| LIMS2 | TB vs ALL | 0 | 0.526 [0.401 - 0.650] | 6.93E-01 | 7.78E-01 | 1.0 | 1.2 | 1.0 | 1.0 |
| FCGR1A | TB vs ALL | -0.14 | 0.522 [0.381 - 0.664] | 7.80E-01 | 8.42E-01 | 478.3 | 269.0 | 900.7 | 428.0 |
| RIPK2 | KD vs ALL | 0.12 | 0.519 [0.351 - 0.686] | 8.33E-01 | 8.65E-01 | 38.5 | 37.6 | 44.5 | 52.4 |
| FCGR1C | TB vs ALL | 0.11 | 0.498 [0.355 - 0.641] | 9.85E-01 | 9.85E-01 | 827.9 | 523.4 | 1415.3 | 807.2 |
| CBX2 | TB vs ALL | 0 | 0.491 [0.428 - 0.554] | 7.96E-01 | 8.48E-01 | 1.0 | 1.0 | 1.0 | 1.0 |
| CDH23 | DB vs ALL | -0.56 | 0.489 [0.344 - 0.633] | 8.74E-01 | 8.96E-01 | 1.0 | 1.0 | 2.0 | 3.1 |
| SGSM3 | KD vs ALL | 0.22 | 0.489 [0.289 - 0.690] | 9.08E-01 | 9.19E-01 | 21.5 | 18.4 | 24.7 | 24.1 |
| ARL3 | DV vs ALL | 0 | 0.485 [0.378 - 0.593] | 7.71E-01 | 8.42E-01 | 1.0 | 1.0 | 1.0 | 1.0 |
| C11ORF82 | KD vs ALL | -0.18 | 0.481 [0.298 - 0.665] | 8.33E-01 | 8.65E-01 | 16.2 | 6.9 | 7.4 | 5.5 |
| CMTM1 | DB vs ALL | -0.18 | 0.478 [0.333 - 0.623] | 7.70E-01 | 8.42E-01 | 105.9 | 80.4 | 189.0 | 101.0 |
| SPATA2L | KD vs ALL | 0 | 0.467 [0.436 - 0.499] | 3.37E-01 | 4.61E-01 | 1.0 | 1.0 | 1.0 | 1.0 |
| LIMS2 | KD vs ALL | 0 | 0.460 [0.319 - 0.600] | 5.71E-01 | 6.69E-01 | 1.0 | 1.2 | 1.0 | 1.0 |
| C10ORF33 | KD vs ALL | 0 | 0.457 [0.351 - 0.562] | 5.16E-01 | 6.32E-01 | 1.0 | 1.0 | 1.0 | 1.0 |
| STRN | DB vs ALL | -0.07 | 0.457 [0.313 - 0.602] | 5.62E-01 | 6.69E-01 | 132.7 | 111.4 | 160.2 | 161.6 |
| HESX1 | KD vs ALL | 0 | 0.439 [0.321 - 0.557] | 3.59E-01 | 4.83E-01 | 1.0 | 1.5 | 1.0 | 1.0 |
| KCTD14 | KD vs ALL | 0 | 0.426 [0.381 - 0.471] | 1.32E-01 | 2.00E-01 | 1.0 | 1.0 | 1.0 | 1.0 |

## Table S5

*Table S5: AUC, sensitivity and specificity of the 3-transcript TB signature in training, test and validation sets.*

|  | AUC | Sensitivity | Specificity |
| --- | --- | --- | --- |
| Training set | 0.884 [0.839-0.930] | 0.761 [0.671-0.851] | 0.882 [0.822-0.933] |
| Test set | 0.879 [0.791-0.966] | 0.957 [0.870-1.000] | 0.676 [0.500-0.824] |
| Validation set | 0.928 [0.872-0.985] | 0.886 [0.771-0.971] | 0.859 [0.766-0.938] |

## Table S6

*Table S6: Confusion matrices for the individual and combined MOVA-models and the multiclass model, with in-sample error rate as a percentage. For the individual MOVA-models the predicted diseases are either "Case" (e.g. DB) or "Other" (e.g. not DB).*

|  |  |  | Predicted disease | | | |  |
| --- | --- | --- | --- | --- | --- | --- | --- |
|  |  | Actual disease | Case | | Other | | In-sample error rate |
| MOVA-model: Individual models | DB vs other | DB | 17 | | 6 | | 18.7% |
|  |  | Not DB | 8 | | 44 | |  |
|  | DV vs other | DV | 16 | | 4 | | 13.3% |
|  |  | Not DV | 6 | | 49 | |  |
|  | KD vs other | KD | 14 | | 0 | | 4.0% |
|  |  | Not KD | 3 | | 58 | |  |
|  | TB vs other | TB | 17 | | 1 | | 2.7% |
|  |  | Not TB | 1 | | 56 | |  |
| Full MOVA-model | |  | DB | DV | KD | TB | 13.3% |
|  |  | C | 1 | 8 | 1 | 6 |  |
|  |  | DB | 16 | 4 | 3 | 0 |  |
|  |  | DV | 0 | 19 | 0 | 1 |  |
|  |  | KD | 1 | 0 | 13 | 0 |  |
|  |  | TB | 0 | 1 | 0 | 17 |  |
|  | | | | | | | |
| Multiclass model | | C | 6 | 2 | 0 | 8 | 0.0% |
|  |  | DB | 23 | 0 | 0 | 0 |  |
|  |  | DV | 0 | 20 | 0 | 0 |  |
|  |  | KD | 0 | 0 | 15 | 0 |  |
|  |  | TB | 0 | 0 | 0 | 18 |  |

# References

1. Herberg JA, Kaforou M, Wright VJ, et al. Diagnostic Test Accuracy of a 2-Transcript Host RNA Signature for Discriminating Bacterial vs Viral Infection in Febrile Children. *JAMA* 2016; **316**(8): 835-45.

2. Anderson ST, Kaforou M, Brent AJ, et al. Diagnosis of childhood tuberculosis and host RNA expression in Africa. *N Engl J Med* 2014; **370**(18): 1712-23.

3. Martinon-Torres F, Salas A, Rivero-Calle I, et al. Life-threatening infections in children in Europe (the EUCLIDS Project): a prospective cohort study. *Lancet Child Adolesc Health* 2018; **2**(6): 404-14.

4. McCrindle BW, Rowley AH, Newburger JW, et al. Diagnosis, Treatment, and Long-Term Management of Kawasaki Disease: A Scientific Statement for Health Professionals From the American Heart Association. *Circulation* 2017; **135**(17): e927-e99.

5. Kampmann B, Seddon JA, Paton J, et al. Evaluating UK National Guidance for Screening of Children for Tuberculosis. A Prospective Multicenter Study. *Am J Respir Crit Care Med* 2018; **197**(8): 1058-64.

6. Du P, Kibbe WA, Lin SM. lumi: a pipeline for processing Illumina microarray. *Bioinformatics* 2008; **24**(13): 1547-8.

7. Schmid R, Baum P, Ittrich C, et al. Comparison of normalization methods for Illumina BeadChip HumanHT-12 v3. *BMC Genomics* 2010; **11**: 349.

8. Ritchie ME, Phipson B, Wu D, et al. limma powers differential expression analyses for RNA-sequencing and microarray studies. *Nucleic Acids Res* 2015; **43**(7): e47.

9. Coin LJ. FSPLS. 0.5.1 ed; 2018. p. fspls: Minimal TB biomarkers.

10. NanoString Technologies. Gene Expression Data Analysis Guidelines 2017. https://university.nanostring.com/gene-expression-data-analysis-guidelines?next=%2Fgene-expression-data-analysis-guidelines%2F894768 (accessed 03/01/2024).

11. Benjamini Y, Hochberg Y. Controlling the False Discovery Rate: A Practical and Powerful Approach to Multiple Testing. *Journal of the Royal Statistical Society Series B (Methodological)* 1995; **57**(1): 289-300.

12. Friedman J, Hastie T, Tibshirani R. Regularization Paths for Generalized Linear Models via Coordinate Descent. *J Stat Softw* 2010; **33**(1): 1-22.

13. Roe JK, Thomas N, Gil E, et al. Blood transcriptomic diagnosis of pulmonary and extrapulmonary tuberculosis. *JCI Insight* 2016; **1**(16): e87238.

14. Wright VJ, Herberg JA, Kaforou M, et al. Diagnosis of Kawasaki Disease Using a Minimal Whole-Blood Gene Expression Signature. *JAMA Pediatr* 2018; **172**(10): e182293.

15. Pennisi I, Rodriguez-Manzano J, Moniri A, et al. Translation of a Host Blood RNA Signature Distinguishing Bacterial From Viral Infection Into a Platform Suitable for Development as a Point-of-Care Test. *JAMA Pediatr* 2021; **175**(4): 417-9.
