# Supplementary material for: Integration and validation of host transcript signatures, including a novel 3-transcript tuberculosis signature, to enable one-step multiclass diagnosis of childhood febrile disease
Source: J Transl Med. 2024 Aug 29;22:802. doi: 10.1186/s12967-024-05241-4 (PMC11360490; doi:10.1186/s12967-024-05241-4)
Supplement: Supplementary file 2 — Additional file 2: Annex 1. Consortia members. [file 12967_2024_5241_MOESM2_ESM.docx]

ANNEX 1: CONSORTIA MEMBERS

Contents

[The UK Kawasaki Disease Genetics consortium: 1](#_Toc155190582)

[GENDRES www.gendres.org: 2](#_Toc155190583)

[EUCLIDS www.euclids-project.eu: 4](#_Toc155190584)

[ILULU 6](#_Toc155190585)

# The UK Kawasaki Disease Genetics consortium:

Section of Paediatric Infectious Disease, Imperial College London; Professor M Levin, Rachel Galassini, Dr Victoria Wright, Dr Jethro Herberg; Addenbrookes Hospital, Cambridge: Dr Y Singh (PI), J Bytham, J Sharp; Airedale General Hospital Dr P Bala (PI), A Kitching; Alder Hey children’s Hospital: Dr S Paulus (PI), Prof E Carol (PI), Dr B Larru (PI). S Wadeson, J Johnstone, R Jennings; Birmingham Children’s Hospital; Dr A Chickermane (PI), K Cotter; Bradford Royal Infirmary: Dr H Jepps (PI); T Booth, R Swingler; Bristol Royal Infirmary: Prof R Tulloh (PI); Karen Sheehan; Burton Hospital: Dr M Ahmed (PI), S Boswell, C Backhouse; Calderdale Royal Hospital Dr M Olabi (PI), KU Rahman (PI), S Kilroy, M Home; Durham & Darlington NHS Trust: Dr T Banerjee (PI), Dr G Nyamugunduru (PI), A Cowton, D Egginton; East Surrey Hospital; Dr M Jawad (PI)L Bailey; Evelina Children’s Hospital: Dr E Menson (PI); Great Ormond Street Hospital, Dr P Brogan (PI), Y Glackin; Harrogate Hospital: Miss C Brunskill (PI); Heartlands Hospital Dr S Hackett (PI), J Daglish; Hereford County Hospital Dr S Meyrick (PI), E Collins; Hull University Teaching Hospital, Mr D Bolton (PI); Imperial College Healthcare NHS Trust; Dr J Herberg (PI), S Gormley, S Mustafa; Ipswich Hospital: Dr P Desai (PI) L Hunt; Kingston Hospital Dr T Chawatama (PI), Dr S Luck (PI), J Crooks, T O’Brien; Leeds General Infirmary Dr S O’Riordan (PI), N Balatoni, N Maher; Macclesfield General Hospital: Dr Chandrasekaran (PI), N Keenan; New Cross Hospital, Wolverhampton, Dr K Davies (PI), S Kempson, C Busby; North Manchester General Hospital: Dr E Odeka (PI), G O’Connor; North Tees & Hartlepool Dr I Haar (PI), G Osborne, H Walker; Northwick Park Hospital: Dr A Williams (PI); Oldham Hospital Dr E Odeka (PI), L Woodward, C Rishton; Peterborough City Hospital; Dr V Puthi (PI), A Pearson, P Goodyear; Pinderfields General Hospital, Dr C Davidson (PI), Dr N De Vere (PI), G Castle; Royal Albert Edward Infirmary, Dr M Farrier (PI), N Pemberton; Royal Bolton Hospital: Dr S Misra (PI), C Fish, P Graham, J Henry; Royal Lancaster Infirmary, Dr A Olabi (PI) K Allison; Royal Shrewsbury Hospital: Dr A Kannivelu (PI), Mr J Jones (PI); Royal Stoke University Hospital: Dr J Alexander (PI) E Roe, R Pringle, A Cope; Sheffield Children’s Hospital, Dr F Shackley (PI), S Gormley; South Tees Hospital: Dr R Kumar (PI), GMcilhinney, S Armstrong; St George’s Hospital, Tooting, Prof P Heath (PI), E Vitale, J Stuart; St Richard’s Hospital, Chichester: Dr N Brennan (PI), S Floyd; Stepping Hill Hospital, Stockport, Dr C Cooper, S Bennett; Tameside Hospital, Dr A Petkar (PI), Dr C Greenway (PI), W Hulse; The Royal Alexandra Hospital, Brighton; Dr K Fidler (PI), K Moscovici, S Sobowieck Kouman; The Royal Brompton Hospital, Dr F Franklin (PI), Dr M Bartsota; The Royal Cornwall Hospital, Dr N Venkata (PI), Dr A Prendiville (PI), Dr O Elmasry (PI), Mrs H Osborne (PI), G Craig, B Bromage; Torbay Hospital, Dr M Raman (PI), Ms P Fitzell (PI), H Bearne, J Palmer; UK Kawasaki Support Group S Davidson, N Clements

# GENDRES:

**Website**: www.gendres.org

Federico Martinón-Torres, Miriam Cebey López, Antonio Salas Ellacuriaga, Ana Vega Gliemmo, José Peña Guitián, Alexa Regueiro, Antonio Justicia Grande, Leticia Pías Peleteiro, María López Sousa, María Jose de Castro, Carmen Curros Novo, Elena Rodrigo, Miriam Puente Puig, Rosaura Leis Trabazo, Nazareth Martinón Torres, Alberto Gómez Carballa, Jacobo Pardo Seco, Sara Pischedda, José María Martinón Sánchez, Belén Mosquera Pérez, Isabel Villanueva González, Lorenzo Redondo Collazo, Carmen Rodríguez-Tenreiro, María del Sol Porto Silva and Federico Martinón Torres (Área Asistencial Integrada de Pediatría and GENVIP, Hospital Clínico Universitario, Santiago de Compostela); Máximo Francisco Fraga Rodríguez, Orlando Fernández Lago, José Ramón Antúnez (Biobank, Servicio Anatomía Patológica, Hospital Clínico Universitario, Santiago de Compostela); Enrique Bernaola Iturbe, Laura Moreno Galarraga, Jorge Álvarez, Mercedes Herranz, Francisco Gil, Eva Gembero, Jorge Rodríguez (Hospital Materno Infantil Virgen del Camino, Pamplona); Teresa González López, Delfina Suarez Vázquez, Ángela Vázquez Vázquez, Susana Rey García, Nathalie Carreira Sande, Ana López Fernández, Nuria Romero Pérez (Complejo Hospitalario Universitario de Orense); José Antonio Couceiro Gianzo, Nazareth Fuentes Perez (Complejo Hospitalario Universitario de Pontevedra); Francisco Giménez Sánchez, Miguel Sánchez Forte (Hospital Torrecárdenas, Almería); Cristina Calvo Rey, María Luz García García, Iciar Olabarrieta Arnal, Adelaida Fernández Rincón (Hospital Severo Ochoa de Madrid); Ignacio Oulego Erroz, David Naranjo Vivas, Santiago Lapeña, Paula Alonso Quintela, Jorge Martínez Sáenz de Jubera, Estibaliz Garrido García (Hospital de León); Ana Grande Tejada (Hospital Materno Infantil de Badajoz); Cristina Calvo Monge, Eider Oñate Vergara (Hospital de Donostia, San Sebastián); Jesús de la Cruz Moreno, Mª Carmen Martínez Padilla, Eugenia Villanueva Martínez, Ana González Espín, María de las Mercedes Martínez Rebollo, María Rocío Martín Moya (Complejo Hospitalario de Jaén); Manuel Baca Cots (Hospital Quirón, Málaga), David Moreno Pérez, Ana Cordón Martínez, Antonio Urda Cardona, José Miguel Ramos Fernández, Esmeralda Núñez Cuadros (Hospital Carlos Haya, Málaga); Susana Beatriz Reyes, María Cruz León León, Santiago Alfayate (Hospital Virgen de la Arrixaca, Murcia); Cristina Calvo, Carlos Grasa, Cristian Quintana Ortega, Leticia La Banda Montalvo, María Lopez Cerdán, Ana Dominguez Castells (Hospital Universitario La Paz, Madrid); Francisco Giménez Sánchez (Hospital Inmaculada); Andrés J. Alcaraz Romero, Diego Bautista Lozano, Sara Uillen Martin (Hospital Universitario de Getafe); Roi Piñeiro (Hospital General de Villalba); Juan Ignacio Sánchez Díaz, Alba Palacios Cuesta (Hospital 12 Octubre); Elvira González Salas, Sira Fernández De Miguel (Hospital Clínico de Salamanca); Belen Joyanes Abancens, Esther Aleo Lujan (Hospital clínico San Carlos); Alfredo Tagarro García, María Luisa Herreros, Rut del Valle, Libertad Latorre Navarro (Hospital Universitario Infanta Sofía); María Concepción Zazo Sanchidrián, Mariano Esteban, Marta González Lorenzo, Mª Carmen Vicent Castello (Hospitcristial General Universitario de Alicante); Lorena Moreno Requena, Juan Luis Santos Pérez (Complejo Hospitalario Universitario de Granada); César Gavilán Martín, Lucía González-Moro Azorín (Hospital Universitario de San Juan de Alicante); Monterrat López Franco, Manuel Silveira Cancela (Hospital de Burela); María José Cilleruelo Ortega, Luz Golmayo, Francisca Portero Azorín (Hospital Universitario Puerta de Hierro-Majadahonda); Andrés Concha Torre, Lucía Rodríguez García (Hospital Universitario Central de Asturias); Carlos Rodrigo Gonzalo de Liria, Andrés Antón Pagarolas (Hospital Vall d'Hebron); Maria Méndez, Critina Prat (Hospital Germans Trias i Pujol); Jesús López-Herce, Miriam García Samprudencio, Gema Manrique Martín, Paula García Casas, Débora Sanz Alvarez (Hospital General Universitario Gregorio Marañón); María Jesús Cabero (Hospital Valdecilla); Miguel Lillo Lillo, Marta Pareja (Hospital General de Albacete); Pablo Rojo, Cristina Epalza (Hospital 12 Octubre); Adriana Navas Carretero, Estefanía Barral, Miriam Herrera (Hospital Infanta Leonor); Elvira Cobo Vazquez (Hospital Universitario Fundación de Alcorcón); Elena del Castillo Navío (Hospital Materno Infantil de Badjoz); Patricia Flores Perez (Hospital del Niño Jesús); Paula García Casas (Hospital Ramón y Cajal); Esteban Gómez Sanchez, Juan Valencia Ramos (Hospital Universitario de Burgos); Francisco Javier Pilar Orive, Elva Rodriguez Merino (Hospital Univeristario Cruces); Ana Pérez Aragón (HMI Virgen de las Nieves de Granada); Mª Yolanda Ruiz del Prado (Hospital San Pedro); David Moreno, Beatriz Carazo (Hospital Carlos Haya); Jordi Antón (Hospital Sant Joan de Déu); María teresa Rives Ferreiro (Hospital Universitario de Navarra).

# EUCLIDS:

**Website**: www.euclids-project.eu

Michael Levin, Lachlan Coin, Stuart Gormley, Shea Hamilton, Jethro Herberg, Bernardo Hourmat, Clive Hoggart, Myrsini Kaforou, Vanessa Sancho-Shimizu, Victoria Wright, Amina Abdulla, Paul Agapow, Maeve Bartlett, Evangelos Bellos, Hariklia Eleftherohorinou, Rachel Galassini, David Inwald, Meg Mashbat, Stephanie Menikou, Sobia Mustafa, Simon Nadel, Rahmeen Rahman, Hannah Shailes, Clare Thakker, S Bokhandi, Sue Power, Heather Barham, N Pathan, Jenna Ridout, Deborah White, Sarah Thurston, Saul Faust , Sanjay Patel, Jenni McCorkell, P Davies , Lindsey Crate, Helen Navarra, Stephanie Carter, R Ramaiah , Rekha Patel, Catherine Tuffrey , Andrew Gribbin, Sharon McCready, Mark Peters, Katie Hardy, Fran Standing, Lauren O’Neill, Eugenia Abelake, Akash Deep, Eniola Nsirim, Andrew Pollard, Louise Willis, Zoe Young, C Royad, Sonia White, PM Fortune, Phil Hudnott, Federico Martinón-Torres, Antonio Salas, Fernando Álvez González, Ruth Barral-Arca, Miriam Cebey- López, María José Curras-Tuala, Natalia García, Luisa García Vicente, Alberto Gómez-Carballa, Jose Gómez Rial, Andrea Grela Beiroa, Antonio Justicia Grande, Pilar Leboráns Iglesias , Alba Elena Martínez Santos, Federico Martinón -Torres, Nazareth Martinón-Torres, José María Martinón Sánchez, Beatriz Morillo Gutiérrez, Belén Mosquera Pérez, Pablo Obando Pacheco, Jacobo Pardo-Seco, Sara Pischedda, Irene Rivero-Calle, Carmen Rodríguez-Tenreiro, Lorenzo Redondo-Collazo, Sonia Serén Fernández, María del Sol Porto Silva, Ana Vega, Lucía Vilanova Trillo, Susana Beatriz Reyes, María Cruz León León, Álvaro Navarro Mingorance, Xavier Gabaldó Barrios, Eider Oñate Vergara, Andrés Concha Torre, Ana Vivanco, Reyes Fernández, Francisco Giménez Sánchez, Miguel Sánchez Forte, Pablo Rojo, J.Ruiz Contreras, Alba Palacios , Cristina Epalza Ibarrondo, Elizabeth Fernández Cooke, Marisa Navarro, Cristina Álvarez Álvarez, María José Lozano, Eduardo Carreras, Sonia Brió Sanagustín, Olaf Neth, Ma del Carmen Martínez Padilla, Luis Manuel Prieto Tato, Sara Guillén, Laura Fernández Silveira, David Moreno, Ronald de Groot , A.M. Tutu van Furth , Michiel van der Flier, N.P. Boeddha , G.J.A. Driessen , J.A. Hazelzet, T.W. Kuijpers , D. Pajkrt, E.A.M. Sanders, D. van de Beek , A. van der Ende, H.L.A. Philipsen, A.O.A. Adeel , M.A. Breukels , D.M.C. Brinkman , C.C.M.M. de Korte , E. de Vries , W.J. de Waal , R. Dekkers , A. Dings-Lammertink , R.A. Doedens , A.E. Donker , M. Dousma, T.E. Faber , G.P.J.M. Gerrits, J.A.M. Gerver , J. Heidema , J. Homan-van der Veen , M.A.M. Jacobs , N.J.G. Jansen , P. Kawczynski , K. Klucovska , M.C.J. Kneyber , Y. Koopman-Keemink , V.J. Langenhorst , J. Leusink , B.F. Loza , I.T. Merth , C.J. Miedema , C. Neeleman , J.G. Noordzij , C.C. Obihara , A.L.T. van Overbeek – van Gils , G.H. Poortman ,S.T. Potgieter, J. Potjewijd , P.P.R. Rosias , T. Sprong , G.W. ten Tussher , B.J. Thio , G.A. Tramper-Stranders , M. van Deuren , H. van der Meer , A.J.M. van Kuppevelt , A.M. van Wermeskerken , W.A. Verwijs , T.F.W. Wolfs, Luregn J Schlapbach, Philipp Agyeman, Christoph Aebi, Christoph Berger, Eric Giannoni, Martin Stocker, Klara M Posfay-Barbe, Ulrich Heininger, Sara Bernhard-Stirnemann, Anita Niederer-Loher, Christian Kahlert, Paul Hasters, Christa Relly, Walter Baer, Enitan D Carrol, Stéphane Paulus,MHannah Frederick, Rebecca Jennings, Joanne Johnston, Rhian Kenwright, Colin G Fink, Elli Pinnock, Marieke Emonts, Rachel Agbeko, Suzanne Anderson, Fatou Secka, Kalifa Bojang, Isatou Sarr, Ngange Kebbeh, Gibbi Sey, Momodou Saidykhan, Fatoumata Cole, Gilleh Thomas, Martin Antonio, Werner Zenz, Daniela S. Kohlfürst, Alexander Binder, Nina A. Schweintzger, Manfred Sagmeister, Hinrich Baumgart, Markus Baumgartner, Uta Behrends, Ariane Biebl, Robert Birnbacher, Jan-Gerd Blanke, Carsten Boelke, Kai Breuling, Jürgen Brunner, Maria Buller, Peter Dahlem, Beate Dietrich, Ernst Eber, Johannes Elias, Josef Emhofer, Rosa Etschmaier, Sebastian Farr, Ylenia Girtler, Irina Grigorow, Konrad Heimann, Ulrike Ihm, Zdenek Jaros, Hermann Kalhoff, Wilhelm Kaulfersch, Christoph Kemen, Nina Klocker, Bernhard Köster, Benno Kohlmaier, Eleni Komini, Lydia Kramer, Antje Neubert, Daniel Ortner, Lydia Pescollderungg, Klaus Pfurtscheller, Karl Reiter, Goran Ristic, Siegfried Rödl, Andrea Sellner, Astrid Sonnleitner, Matthias Sperl, Wolfgang Stelzl, Holger Till, Andreas Trobisch, Anne Vierzig, Ulrich Vogel, Christina Weingarten, Stefanie Welke, Andreas Wimmer, Uwe Wintergerst, Daniel Wüller, Andrew Zaunschirm, Ieva Ziuraite, Veslava Žukovskaja, Martin L. Hibberd, Sonia Davila, Isabel Delany

# ILULU

Institute of Infectious Diseases and Molecular Medicine, University of Cape Town Nonzwakazi Bangani, Lizl Bashe, Melina Carr, Hannah P. Gideon, Rene Goliath, Yekiwe Hlombe, Vanessa January, Bekekile Kwaza, Suzaan Marais, Marc Mendelson, Tolu Oni, Fadheela Patel, Ronnett Seldon, Relebohile Tsekela, Katalin A. Wilkinson, Robert J. Wilkinson, Kathryn Wood; London School of Hygiene & Tropical Medicine/Karonga Prevention Study Lyn Ambrose, Amelia C. Crampin, Hazel M. Dockrell, Neil French, Lumbani Munthali, Bagrey Ngwira, Amos Phiri, Femia Zgambo; Red Cross War Memorial Children's Hospital, University of Cape Town Margaret Cooper, Brian Eley, Mabel Gcuwa, Spasina King, Glynis Kossew, Karen McCabe, Wonita Petersen, Sandra Pienaar, Vashini Pillay; Liverpool School of Tropical Medicine/Malawi-Liverpool-Wellcome Trust Clinical Research Programme, University of Malawi College of Medicine Benjamin Allubha, George Chagaluka, Angeziwa Chunga, Janet Dube, Robert S. Heyderman, Annie Joabe, Martha Kalemba, Anne Kerr, Monica Matola, Rachel Mlotha, Agnes Mwale, David Mzinza; Brighton and Sussex Medical School, University of Sussex Suzanne T. Anderson, Gillian Baker, Claire M. Banwell, Terry Bishop, Natalie Chaplin, Julian Golland, Florian Kern, Susan Poore, Jayne Wellington; Imperial College London Andrew J. Brent, Lachlan J. Coin, Hariklia Eleftherohorinou, Melissa S. Hamilton, Myrsini Kaforou, Paul R. Langford, Michael Levin, Stephanie Menikou, Victoria J. Wright.
